# Human repair‐related Schwann cells adopt functions of antigen‐presenting cells in vitro

**DOI:** 10.1002/glia.24257

**Published:** 2022-08-17

**Authors:** Jakob Berner, Tamara Weiss, Helena Sorger, Fikret Rifatbegovic, Max Kauer, Reinhard Windhager, Alexander Dohnal, Peter F. Ambros, Inge M. Ambros, Kaan Boztug, Peter Steinberger, Sabine Taschner‐Mandl

**Affiliations:** ^1^ St. Anna Children's Cancer Research Institute (CCRI) Vienna Austria; ^2^ St. Anna Children's Hospital Vienna Austria; ^3^ Department of Plastic, Reconstructive and Aesthetic Surgery Medical University of Vienna; ^4^ Department of Orthopedics and Trauma Surgery Medical University of Vienna Vienna Austria; ^5^ Ludwig Boltzmann Institute for Rare and Undiagnosed Diseases (LBI‐RUD) Vienna Austria; ^6^ Center for Molecular Medicine (CeMM) Vienna Austria; ^7^ Institute of Immunology Medical University of Vienna Vienna Austria

**Keywords:** antigen‐presenting cell, immunocompetence, immunoregulatory, inflammation, nerve injury, neuropathies, PD‐L1, Schwann cell

## Abstract

The plastic potential of Schwann cells (SCs) is increasingly recognized to play a role after nerve injury and in diseases of the peripheral nervous system. Reports on the interaction between immune cells and SCs indicate their involvement in inflammatory processes. However, the immunocompetence of human SCs has been primarily deduced from neuropathies, but whether after nerve injury SCs directly regulate an adaptive immune response is unknown. Here, we performed comprehensive analysis of immunomodulatory capacities of human repair‐related SCs (hrSCs), which recapitulate SC response to nerve injury in vitro. We used our well‐established culture model of primary hrSCs from human peripheral nerves and analyzed the transcriptome, secretome, and cell surface proteins for pathways and markers relevant in innate and adaptive immunity, performed phagocytosis assays, and monitored T‐cell subset activation in allogeneic co‐cultures. Our findings show that hrSCs are phagocytic, which is in line with high MHCII expression. Furthermore, hrSCs express co‐regulatory proteins, such as CD40, CD80, B7H3, CD58, CD86, and HVEM, release a plethora of chemoattractants, matrix remodeling proteins and pro‐ as well as anti‐inflammatory cytokines, and upregulate the T‐cell inhibiting PD‐L1 molecule upon pro‐inflammatory stimulation with IFNγ. In contrast to monocytes, hrSC alone are not sufficient to trigger allogenic CD4^+^ and CD8^+^ T‐cells, but limit number and activation status of exogenously activated T‐cells. This study demonstrates that hrSCs possess features and functions typical for professional antigen‐presenting cells in vitro, and suggest a new role of these cells as negative regulators of T‐cell immunity during nerve regeneration.

## Main Points

- Human repair‐related Schwann cells (hrSC) function as professional antigen‐presenting cells.
- HrSCs up‐regulate PD‐L1 upon pro‐inflammatory IFNγ stimulation.
- HrSCs hamper CD^4+^ and CD^8+^ T‐cell activation.

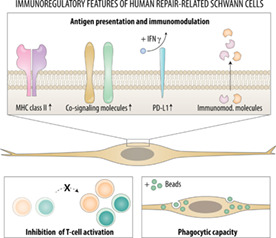

## 1 INTRODUCTION

Schwann cells (SCs) are glial cells of the peripheral nervous system and possess capacities that go far beyond the preservation of axon integrity. Upon nerve injury, SCs undergo extensive morphological and expression changes and acquire distinct repair features in a process referred to as “adaptive cellular reprogramming” (Gomez‐Sanchez et al., 2017; Jessen & Mirsky, 2016; Weiss et al., 2016). In this dedicated repair cell state, SCs re‐enter the cell cycle and execute specialized functions to coordinate the multistep process of nerve regeneration, such as the recruitment of immune cells, the breakdown of myelin debris, remodeling of the extracellular matrix, and the expression of neurotrophic and neuritogenic factors for axon survival, regrowth, and guidance (Gomez‐Sanchez et al., 2015; Jang et al., 2016; Jessen & Mirsky, 2016; Nocera & Jacob, 2020; Tofaris et al., 2002; Weiss et al., 2016). Moreover, numerous studies support that the highly adaptive cellular state of SCs plays a role in pathological conditions such as neuropathies and tumor development (Azam & Pecot, 2016; Bunimovich et al., 2017; Direder et al., 2021; Weiss et al., 2021). We have shown that tumor‐associated SCs in neuroblastic tumors adopt a similar phenotype as upon nerve injury and exert anti‐proliferative and pro‐differentiating effects through the release of until then unknown neurotrophins, such as EGFL8 (Ingeborg M. Ambros et al., 1996; Weiss et al., 2021). As knowledge on the involvement of SCs during regeneration and pathologies is continuously expanding, their immunomodulatory potential gains increasing interest (Armati et al., 1990; Hörste et al., 2008; Zhang et al., 2020). Schwann cells have been demonstrated as immune competent cells that contribute to inflammatory and hereditary neuropathies Meyer zu Hörste et al., 2008). However, less is known about the impact of human SCs on the inflammatory processes during peripheral nerve regeneration (Bergsteinsdottir et al., 1991; Rutkowski et al., 1999; Toews et al., 1998; Weiss et al., 2016).

Similar to any injury site in the body, injured nerves experience an early pro‐inflammatory response by the influx of immune cells that is followed by termination of the immune response to allow tissue regeneration. Previous studies showed that SCs secrete a variety of cytokines and chemokines which attract monocytes and neutrophils to the site of nerve injury (Bergsteinsdottir et al., 1991; Rutkowski et al., 1999; Tofaris et al., 2002). Their expression could be partially mediated by axon derived molecules recognized by SCs via toll‐like receptors (TLRs) (Goethals et al., 2010; Kaisho & Akira, 2000; Karanth et al., 2006; Lee et al., 2006; Meyer Zu Horste et al., 2010; Meyer zu Hörste et al., 2008). Moreover, endoneural macrophages within injured nerves were shown to adapt a specialized regenerative phenotype with neuroprotective and pro‐angiogenic capacities and express proteins associated with an anti‐inflammatory profile (Cattin et al., 2015; Ydens et al., 2012, 2020; Yin et al., 2013). Schwann cells might be involved in polarizing macrophages toward this regenerative phenotype, but so far, the underlying factors remain unknown (Stratton et al., 2016; Stratton & Shah, 2016).

Schwann cells can also interact with T‐cells by expressing major histocompatibility complex class II (MHCII) receptors and co‐signaling molecules (Armati et al., 1990; Hörste et al., 2008; Murata & Dalakas, 2000). However, upregulation of MHCII on SCs was primarily reported in neuropathies (Mancardi et al., 1988; Meyer Zu Horste et al., 2010; Van Rhijn et al., 2000) and upon stimulation with IFNy (Armati et al., 1990; Lilje & Armati, 1997; Samuel et al., 1987), which is a potent inducer of MHCII expression in antigen‐presenting cells (APCs). In contrast, our previous research showed that human repair‐related SCs highly upregulate MHCII in culture independent of IFNy and within nerve explants serving as a human ex vivo injury model (Weiss et al., 2016). Furthermore, these repair‐related SCs expressed genes of co‐signaling molecules, MHCII transcriptional co‐activator *CIITA*, and other molecules involved in the antigen processing and presentation machinery (Weiss et al., 2016), suggesting a biological relevance of MHCII expression in injured human nerves. In line with our findings from ex vivo human nerves (Weiss et al., 2016), an in vivo study comparing the transcriptome of acutely dissected and injured human sural nerves confirmed MHCII upregulation in the injured nerve piece (Welleford et al., 2020).

In contrast to studies on human nerve explants and primary SC cultures, animal studies comparing the transcriptomes of control versus injured sciatic nerves in rats (Yi et al., 2015) and mice (Arthur‐Farraj et al., 2017) did not report an upregulation of MHCII‐associated genes in the injured condition, which indicates species specific differences in the SC response to injury. Indeed, nerve injury conditions and regeneration programs differ between rodents and humans, which is also reflected in a significant translational gap demonstrated by promising animal studies but a lack of clinical therapies for human nerve injuries (Höke, 2006; Meyer Zu Reckendorf et al., 2020; Monje, 2020). A further major difference between rodent and human SCs lies in their capacity to myelinate DRG axons in vitro. The primary human‐cultured SC cannot be stimulated to myelinate DRG axons under conditions that allow myelin formation in rat SCs (Monje et al., 2018; Morrissey et al., 1995). This is why rodents are important models to study different aspects of nerve injury and regeneration, but are not adequate to study immunomodulatory properties found for human SCs. Instead, robust human models need to be employed.

Antigen‐presenting cells function as local modulators of T‐cell response upon inflammatory stimulation. The outcome of this modulation is dependent on the expression of co‐stimulatory or co‐inhibitory surface molecules recognized by T‐cells together with MHCII. Indeed, rodent SCs could be triggered to activate T‐cells by presenting endogenous as well as exogenous antigens (Duan et al., 2007; Kingston et al., 1989; Spierings et al., 2000; Steinhoff & Kaufmann, 1988; Wekerle et al., 1986). Moreover, T‐cell activation through MHCII expressing SCs has been associated with post‐traumatic inflammation and neuropathic pain in diseased peripheral nerves of mice (Hartlehnert et al., 2017). Hence, the SC function as non‐professional APC has mainly focused on the promotion of T‐cell activation resulting in (auto‐) inflammatory or infectious neuropathies, rather than suppression of activated T‐cells. The latter is executed by APCs to restore immune homeostasis and prevent auto‐immunity to self‐proteins. In line with a potential T‐cell inhibiting function of SCs, our previous transcriptomic analyses have indicated that the primary human SCs express genes associated with T‐cell suppression such as *PD‐L1* and *DC‐HIL* (Weiss et al., 2016) but a functional relevance remains to be established.

Based on the increasing body of studies supporting the immunocompetence of SCs and their recognized role in nerve injury and disease (Meyer Zu Horste et al., 2010; Weiss et al., 2021; S. H. Zhang et al., 2020), we here set out to investigate immunoregulatory features of human SCs in an injury condition. To this end, we employ a robust and well characterized in vitro model for human repair SCs. We cultured the primary human SCs and performed phagocytosis assays, analyzed the secretion of immunomodulatory mediators, and profiled their repertoire of co‐signaling molecules as well as upon TLR and inflammatory stimulation. We further assessed the ability of SCs to modulate T‐cell activation and polarization in vitro.

## 2 METHODS

### 2.1 Human material

The collection and research use of human peripheral nerve tissues and human tumor specimen was conducted according to the guidelines of the Council for International Organizations of Medical Sciences and World Health Organization and has been approved by the local ethics committees of the Medical University of Vienna (EK2281/2016 and 1216/2018). Informed consent has been obtained from all patients participating in this study.

Neuroblastoma cell lines and primary cultures are available upon request. Primary Schwann cell cultures and tumor tissues are limited materials and therefore cannot be provided.

### 2.2 Isolation of the primary human Schwann cells

Schwann cells were isolated, cultured and enriched as the previously described (Weiss et al., 2018). Briefly, peripheral nerves were cut into 2–3 cm pieces and nerve fascicles were pulled out of the surrounding epineural tissue. The isolated fascicles were cut into ~0.5 cm pieces and incubated in a digestion solution containing αMEM GlutaMAX™ (Gibco), 10% FCS (PAA), 1% Pen/strep (Pan Biotech), 1 mM sodium pyruvate (Pan Biotech), 25 mM HEPES (Pan Biotech), 0.125% collagenase Type IV (Gibco), 1.25 U/ml Dispase II (Sigma‐Aldrich) and 3 mM CaCl (Sigma‐Aldrich) at 37 °C, for 20 h. The digested tissue was pelleted and resuspended in SC expansion medium (SCEM) containing MEMα, 1% Pen/Strep, 1 mM sodium pyruvate, 25 mM HEPES, 10 ng/ml hu FGF basic (PeproTech), 10 ng/ml hu Heregulin‐β1 (PeproTech), 5 ng/ml hu PDGF‐AA (PeproTech), 0.5% N2 supplement (Gibco), 2 μM forskolin (Sigma‐Aldrich) and 2% FCS. Cells were seeded in 0.01% Poly‐L‐lysine (PLL, Sigma‐Aldrich) and 4 μg/ml laminin (Sigma‐Aldrich) coated culture dishes. Half of the medium was changed twice a week. As passage 0 (p0) cultures consisted of SCs and fibroblast‐like cells, SCs were enriched before experimentation, by exploiting their differential adhesion potential to plastic, described in Weiss et al. (2018). As previously shown, human SCs adopt a repair‐related phenotype in culture (Weiss et al., 2016) and SCs are referred to as human repair‐related SCs (hrSC).

### 2.3 Neuroblastoma cell lines

The used neuroblastoma cell lines (NB cells) are derived from biopsies or surgical resection of aggressively behaving, high‐risk neuroblastomas. In‐house established, low passage NB cell lines STA‐NB‐6, ‐7, ‐10, and ‐15 as well as the cell lines, SH‐SY5Y, IMR5, and CLB‐Ma (kindly provided by Dr Valerie Combaret, Centre Leon Berard, France) (I M Ambros et al., 1997; Biedler et al., 1973; Biedler et al., 1978; Combaret et al., 1995; Fischer & Berthold, 2003; Momoi et al., 1980; Stock et al., 2008) were used for experimentation and cultured in MEMα GlutaMAX™, 1% Pen/Strep, 1 mM sodium pyruvate, 25 mM HEPES, and 10% FCS. NB cells are used in this study as model to reflect neuronal cells.

### 2.4 Primary human T‐cells

For the T‐cell isolation, a buffy coat was obtained from the Austrian Red Cross and diluted 1:4 in 1x PBS. Density gradient centrifugation was performed by transferring the blood onto 20 ml Lymphoprep solution (StemCell Technologies) and centrifugation for 30 min at 400*g* at room temperature (RT) without breaks. Mononuclear cells were carefully removed from the interphase layer and transferred into 50 ml 1x PBS and centrifuged at 300*g* for 10 min. Then, the medium was removed and T‐cells were isolated with the Pan T‐cell Isolation Kit (Miltenyi Biotec) according to the manufacturer's protocol using magnetic activated cell sorting (MACS). Briefly, cells were counted and resuspended in 40 μl of MACS buffer (PBS, pH 7.2, 0.5% bovine serum albumin [BSA], 2 mM EDTA) per 10^7^ cells. For each 40 μl, 10 μl of PAN T‐Cell Biotin Antibody Cocktail (Miltenyi Biotec) was added and incubated for 5 minutes at 4°C. Then, 30 μl of MACS buffer and 20 μl of Pan T‐Cell Microbead Cocktail was added per each 50 μl solution and incubated for additional 10 min at 4°C. For the magentic separation, MACS LS columns (Miltenyi Biotec) were placed in the magnetic field of a MACS separator (Miltenyi Biotech) and the column was rinsed with 3 ml MACS buffer. The cell suspension was added and the flow‐through containing the unlabelled, CD3 positive T‐cells, was collected. Cells were frozen in Cryostor freezing medium (Biolife Solutions) and stored in liquid nitrogen until the day of the experiments.

### 2.5 Isolation of peripheral blood monocytes

For monocyte co‐cultures, monocytes were isolated from healthy donor peripheral blood. Mononuclear cells were obtained as described above. PBMCs were immediately used and monocytes were isolated with a negative selection pan monocyte isolation kit (Miltenyi Biotec) according to the manufacturer's protocol. Briefly, cells were counted and resuspended in 30 μl of MACS buffer (PBS, pH 7.2, 0.5% bovine serum albumin [BSA], 2 mM EDTA) per 10^7^ cells. Further, 10 μL of FcR Blocking Reagent (Miltenyi Biotec) was added. For each 40 μl, 10 μl of PAN Monocyte Biotin Antibody Cocktail (Miltenyi Biotec) was added and incubated for 5 min at 4°C. Then, 30 μl of MACS buffer and 20 μl of Pan Microbead Cocktail was added per each 50 μl solution and incubated for additional 10 min at 4°C. For the magentic separation, MACS LS columns (Miltenyi Biotec) were placed in the magnetic field of a MACS separator (Miltenyi Biotech) and the column was rinsed with 3 ml MACS buffer. The cell suspension was added and the flow‐through containing the unlabelled, CD14 positive monocytes, was collected. After isolation monocytes were immediately used for co‐cultivation experiments.

### 2.6 RNA sequencing and data analysis

RNA‐sequencing datasets have been previously published and are available at the Gene expression omnibus (GEO) repository under the identifiers GSE94035 (MNC, *n* = 5), GSE90711 (SC, *n* = 5), GSE90711 (NB primary cultures, *n* = 5 over 3 patient cultures STA‐NB‐6, STA‐NB‐7, and STA‐NB‐15). RNA isolation, library preparation and sequencing on a Illumna Hiseq 2000 platform were performed as previously described (Weiss et al., 2016, 2021). Short read sequencing data was quality checked using FASTQC (http://www.bioinformatics.babraham.ac.uk/projects/fastqc) and QoRTs (Hartley & Mullikin, 2015) and then aligned to the human genome hs37d5 (ftp://ftp.1000genomes.ebi.ac.uk/) using the STAR aligner (Dobin et al., 2013) yielding a minimum of 11.6 million aligned reads in each sample. Further analysis was performed in the R statistical environment using Bioconductor packages (Gentleman et al., 2004). Count statistics for Ensembl (GRCh37.75) genes were obtained by the “featureCounts” function (package “Rsubread”) and differential expression analysis was performed by edgeR and voom (Ritchie et al., 2015). For differential gene expression analysis only genes passing a cpm (counts per gene per million reads in library) cut‐off of 1 in more than two samples were included. All *p*‐values were corrected for multiple testing by the Benjamini–Hochberg method. Genes with an adjusted *q*‐value <0.05 and a log2 fold change >1 (|log2FC| > 1) were referred to as “significantly regulated” and used for functional annotation analysis via gene set enrichment analysis (GSEA) using MSigDB according to Subramanian et al. (2005) and Mootha et al. (2003).

### 2.7 Phagocytosis assay

5 × 10^4^ enriched p1 hrSCs were seeded per well of an 8‐well chamber slide (Ibidi) coated with PLL/laminin and cultured in SCEM. After 48 h, half of the medium was replaced with fresh SCEM containing 1 μm big carboxylate‐modified polystyrene, fluorescent yellow‐green latex beads (SIGMA‐Aldrich) at a concentration of 8 × 10^6^ beads/well (∼100 beads/cell) for 15 h at 37°C. Thereafter, cells were washed three times with 1x PBS and fixed with Roti‐Histofix (Roth) for 10 min at RT. Cells were stored at 4°C in 1x PBS until multicolor immunofluorescence staining was performed.

### 2.8 Immunofluorescence stainings

All antibody details, dilutions and incubation times are listed in Supplementary Table 1. If not stated otherwise, the staining procedure was performed on RT and a washing step (3 times with 1x PBS for 5 min) was performed after each antibody incubation step, except after permeabilization. For extracellular staining, grown cells were blocked with 1x PBS containing 3% goat serum (DAKO) for 30 min at RT, followed by incubation with antibodies against extracellular targets diluted in 1x PBS containing 1% BSA (Sigma‐Aldrich) and 1% goat serum. Cells were then incubated with the secondary antibodies diluted in 1x PBS containing 1% BSA and 1% goat serum. For permeabilization, cells were exposed to 1x PBS containing 1% BSA, 0.3% Triton‐X (Sigma‐Aldrich) and 3% goat serum for 10 min. Thereafter, cells were incubated with primary antibodies against intracellular targets diluted in 1x PBS containing 1% BSA, 0.1% Triton‐X and 1% goat serum, followed by incubation with secondary antibodies diluted in 1X PBS containing 1% BSA, 0.1% Triton‐X and 1% goat serum. Afterwards, 2 μg/mL 4′,6‐Diamidin‐2‐phenylindol (DAPI, Sigma‐Aldrich) in 1X PBS was added for 2 min followed by a final washing step. Cells were embedded in Fluoromount‐G™ mounting medium (Southern Biotech) and stored at 4°C. Images were taken with a confocal laser scanning microscope (Leica Microsystems, TCS SP8X) using Leica application suite X version 1.8.1.13759 or LAS AF Lite software. Confocal images are depicted as maximum projection of total z‐stacks and brightness and contrast were adjusted in a homogenous manner using the Leica LAS AF software.

### 2.9 FACS characterization of hrSCs

All antibodies used for flow cytometry stainings are listed in Supplementary Table 1. If not stated otherwise, the staining procedure was performed on 4°C. For all phenotyping experiments, cells were cultured in duplicates in each condition. Human repair‐related SCs were cultured in the presence of IFNγ (10^3^ U/mL, Bio‐Techne Ltd.), LPS (10 ng/mL, Sigma‐Aldrich,), Poly:IC (2 μg/mL, Bio‐Techne Ltd.) cross‐linked CD40L (500 ng/mL, Bio‐Techne Ltd.) and IL‐1β (10^4^ U/mL Bio‐Techne Ltd.) for 24 h. Concentrations of cytokines and ligands were chosen according to the manufacturer's recommendations and are in line with previously published levels released by antigen‐presenting cells or lymphocytes in vitro or in peripheral blood plasma (Dillinger et al., 2018). Cells were harvested with Accutase (Sigma‐Aldrich) and transferred into FACS tubes containing 200 μl FACS buffer (0.1% BSA and 0.05% natrium acides in 1x PBS). Cells were washed once with FACS buffer at 1200 rpm for 5 min, resuspended in 50 μl FACS buffer and incubated with 50 μl of an antibody master mix in FACS buffer for 30 min in the dark. Then, cells were washed with FACS buffer and resuspended in 100 μl of Cytofix/Cytoperm solution (BD Biosciences), incubated for 20 min, and again washed with BD 1x perm buffer (BD Biosciences). Next, cells were resuspended in 100 μl 1x perm buffer containing the S100 antibody and incubated for 30 min in the dark. Cells were then washed in 1x perm buffer and resuspended in 100 μl 1x perm buffer with the secondary antibody for 20 min. After a washing step in 1x perm buffer, cells were washed with FACS buffer and resuspended in 100 μl FACS buffer. All samples were measured with a FACSFortessa flow cytometer equipped with 5 lasers (355, 405, 488, 561, and 640 nm) and the FACSDiva software version 8.0 (BD Biosciences) was used.

### 2.10 T‐cell proliferation assay

For all T‐cell experiments, p1 hrSCs, freshly isolated CD14^+^ monocytes and freshly thawed human CD3^+^ T‐cells were used in various conditions. For co‐culture, p1 hrSCs were harvested, counted and seeded at 4 × 10^4^ cells per well in 96 well plates in duplicates. Monocytes were seeded at 4 × 10^4^ cells per well in 96‐well in quadruplicates. T‐cells were thawed, washed once with 1XPBS and centrifuged at 300*g* for 7 min at RT. Cells were counted and labeled with CFSE (Thermo Fisher) at 1 μl/10^7^ cells for 10 min at 37°C. Thereafter, 1 ml FCS buffer was added for 2 min at RT and then washed with αMEM at 300 g for 7 min. Then, cells were FACS sorted for intact cells using a FSC vs SSC gate using the FACS Aria instrument (BD Bioscience). The obtained cells were washed with αMEM at 300*g* for 7 min, counted and 1x10^5^ cells were seeded to the hrSCs or monocytes (co‐culture) or cultured alone (controls) in 96 well plates. For the T‐cell stimulation, 0.25 μl of anti‐CD3/CD28 beads (Gibco) were added. Culture medium containing CD3/CD28 beads was thoroughly replenished every 3 days by one half in 10 day long‐term cultures.

Cells were analyzed via flow cytometry at different time points, that is, at day 2, 4 and 10. Therefore, cells were harvested with Accutase and washed once with FACS buffer. All antibodies used for flow cytometry stainings are listed in Supplementary Table 1. Extracellular staining was performed with 50 μl of antibody mix containing all extracellular antibodies (CD3‐FITC, CD4‐PerCP, CD8APC‐Cy7, and CD25‐PE‐Cy7) in 50 μl of FACS buffer for 30 min at 4°C. Cells were washed and incubated in Fix/Perm solution (Thermo Fisher) at 4°C for 30 min. For the permeabilization, 1x perm buffer (Thermo Fisher) was added and cells were centrifuged at 300*g* for 7 min. Then, the supernatant was discarded and the S100 antibody for intracellular staining was added in 100 μl 1x perm buffer and incubated for 20 min at RT in the dark. After this, cells were washed once with 1x perm buffer, once with FACS buffer and resuspended in 100 μl FACS buffer. For exact quantification of absolute cell numbers, 10 μl AccuCheck Counting Beads (LifeTechnologies) were added to each sample prior to FACS analysis at a FACSFortessa flow cytometer. For data analysis, FACSDiva software version 8.0 was used. Gating for CD4^+^ Th subsets was performed in accordance to Mahnke et al. 2013.

### 2.11 Protein array

The RayBio G‐Series Human Cytokine Antibody Array 4000 Kit (RayBiotech, Inc.) was used to assay secretomes of cell supernatants pooled from 2 independent experiments each from hrSC (*n* = 5), hrSCs co‐cultured with 5 different NB cell cultures (*n* = 5) or NB cell cultures alone (*n* = 5). A total of 274 factors were evaluated (for a complete list of factors, refer to https://www.raybiotech.com/human-cytokine-array-g4000-4/). Arrays were processed according to the manufacturer's instructions. Briefly, protein array membranes were blocked with Blocking Buffer (RayBiotech, Inc.) for 30 min at RT. Membranes were then incubated with 100 μl of undiluted sample for 2 h. After extensive washing with Wash Buffer I and II (RayBiotech, Inc.) to remove unbound materials, the membranes were incubated with biotin‐conjugated antibodies for 2 h at RT. The membranes were then washed and incubated with streptavidin‐fluorescin, again for 2 h at RT, followed by final washing steps. Finally, fluorescence signals were obtained with the GenePix 4000 array scanner (Molecular Devices) using the green channel (Cy3) at an excitation frequency of 532 nm and 700 PMT. The image files generated in this way were aligned to respective .gal files (RayBiotech) and Gene Pix Pro 7 (Molecular Devices) was used to create .gpr files. Each spot was manually inspected on the .gpr file images to ensure accuracy. After background correction and normalization to the internal control, the mean fluorescence intensity (MFI) values were combined for all cell lines and proteins that were differentially expressed (*q* < 0.05) between hrSCs and hrSCs in co‐cultures, compared to neuronal cells as controls, were selected for visualization using the Qlucore Omics Explorer V3.1 software.

### 2.12 Quantification and statistical analysis

If not mentioned otherwise, then statistical analysis was performed with R version 3.4.2 within the R studio interface including publicly available packages CRAN, GGPLOT2, GGBEESWARM and RESHAPE. For pair‐wise comparison paired *t*‐tests were used, for multiple comparisons two‐way ANOVA using a post‐hoc Holm *p*‐value correction was used. *P*‐values of less than 0.05 were considered significant and displayed as *, *p*‐values of less than 0.01 were displayed as **, *p*‐values of less than 0.001 were displayed as ***.

## 3 RESULTS

### 3.1 Human repair‐related Schwann cells show a phagocytic capacity

**FIGURE 1:**
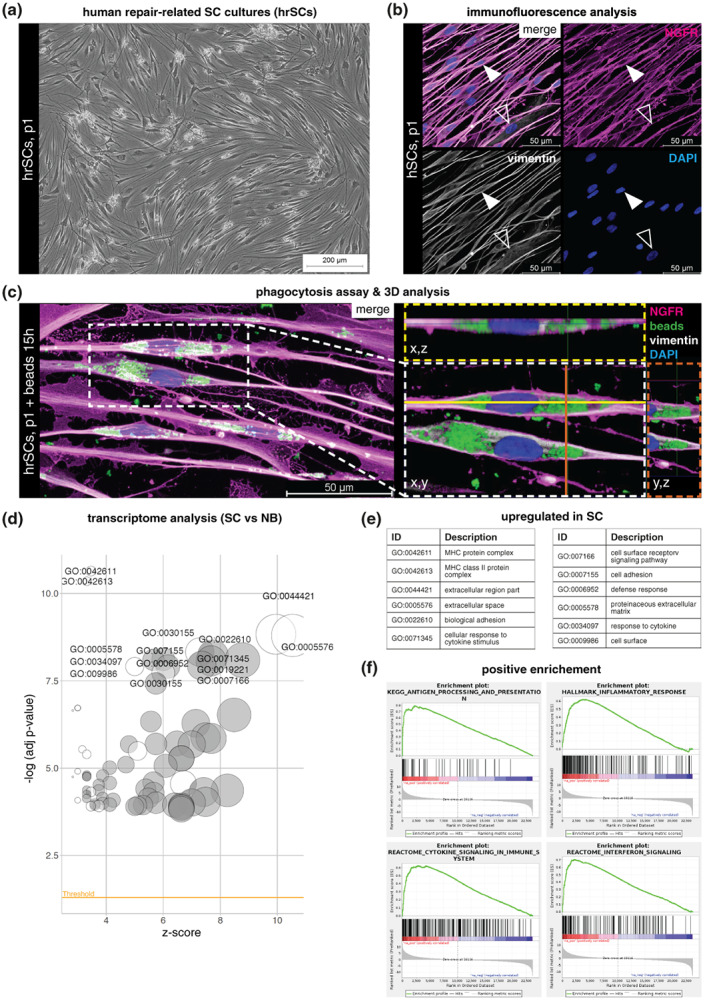
Phagocytosis potential and inflammatory response of hrSCs. (a) Phase contrast image of a representative passage 1 (p1) hrSC culture. (b) Immunostaining of p1 hrSCs for SC marker NGFR (magenta), intermediate filament vimentin (gray) and nuclear stain DAPI (blue); arrowheads indicate a NGFR negative and vimentin positive fibroblast. (c) 3D confocal image analysis of hrSCs exposed to 1 μm in diameter green fluorescent latex beads for 15 h. Cross sections show internalized beads within the SC cytoplasm. (d) Gene ontology (GO) analysis of differentially expressed genes by RNA‐seq between hrSCs (*n* = 5) and NB cultures (*n* = 5 independent biological replicates of 3 donors), −log10 of the enrichment *p*‐values (cut‐off <0.05) for filtered GO categories are plotted relative to Z‐scores of average ratios in each category. Circle size represents the fraction of regulated genes per GO term. (e) Top 12 GO terms among genes upregulated in hrSCs vs NB cultures. (f) Gene set enrichment analysis (GSEA) plots of hrSCs compared to NB cultures. Source data are provided in Supplementary Tables 2–4.

APCs are characterized by their phagocytic ability of exogenous material to process and present antigens via MHCII. To evaluate whether human SCs can take‐up material different from myelin, we applied our previously established protocol for the culture of primary SCs from human peripheral nerves (Weiss et al., 2018). As human SCs possess a repair‐like phenotype and perform repair‐associated functions in culture (Weiss et al., 2016), they are referred to as human repair‐related SCs (hrSCs) in the following. The cultured hrSCs showed the typical spindle‐shaped morphology with a swirled parallel alignment (Figure 1A) and were characterized by immunostainings for the SC marker NGFR (also known as TNR16 or p75^NTR^) (Figure 1B). The co‐staining for vimentin, an intermediate filament expressed by SCs and fibroblasts, visualized a straight filament network within the long hrSC processes and a more branched appearance in fibroblasts (Figure 1B). To obtain information about their phagocytic capacity, we challenged the hrSCs with green fluorescent latex beads (1 μm diameter) for 15 h and then stained for NGFR and vimentin. In extension to our previous study, where we showed that SCs are able to internalize single beads along the processes after 2 h (Weiss et al., 2016), 3D confocal image analysis demonstrated that hrSCs were able to phagocytose numerous beads and to accumulate them within the cell body (Figure 1C).

### 3.2 Transcriptome profiling of human repair‐related Schwann cells revealed immunomodulatory gene signatures

The phagocytic capacity of hrSCs toward cell‐extrinsic material prompted us to evaluate whether pathways associated with inflammation and antigen presentation were active in hrSCs. To this end, we interrogated the transcriptome data generated by deep RNA‐sequencing (RNA‐seq) of hrSC cultures (*n* = 5) and of neuroblastic tumor cells (NB cells) (*n* = 5), which represent an established model for neuronal cells broadly used in neuroscience as shown by us and others (Attoff et al., 2020; Dravid et al., 2021; Schikora et al., 2021; Taylor‐Whiteley et al., 2019; Wang et al., 2016; Weiss et al., 2016, 2021). We determined the differentially expressed genes in neuronal NB cells versus repair‐like SCs to obtain GO terms enriched in the latter as basis for further analysis. Comparison of the transcriptomes of hrSCs and NB cells revealed 5822 differentially expressed genes (*q*‐value<0.01, ∣log2FC >1∣), of which 3057 were upregulated and 2754 were down‐regulated in hrSCs (Supplementary Table 2). Subsequent functional annotation analysis of genes unique to hrSCs demonstrated gene ontology (GO) terms prominent in MHC class I and class II protein complexes, cellular response to cytokine stimulus, and cytokine‐mediated signaling pathways (Figure 1D, Supplementary Table 3). These results were supported by a gene set enrichment analysis (GSEA), which confirmed the enrichment of genes associated with antigen processing and presentation alongside with cytokine signaling and an inflammatory response in hrSCs (Figure 1E, Supplementary Table 4). Taken together, transcriptome profiling of hrSCs provides further evidence of cytokine signaling and an MHCII‐mediated immune response after nerve damage.

### 3.3 Human repair‐related SCs express MHCII and the co‐signaling molecules CD40, CD80, CD86, CD58, HVEM, and B7‐H3

**FIGURE 2:**
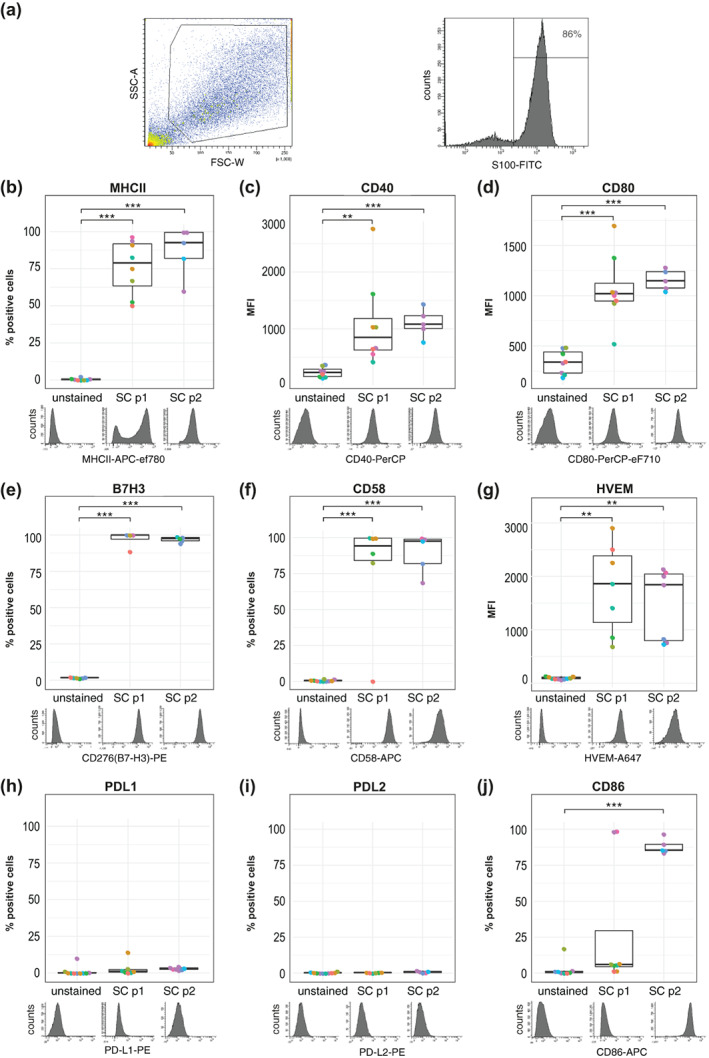
Flow cytometry phenotyping of MHCII and co‐signaling molecules present on human repair‐related Schwann cells. (a) Gating strategy for the identification of S100 positive hrSCs illustrated for one representative experiment. Intact cells are gated in the FSC vs SSC blot and S100 positive cells were selected for further analysis (b–j). Box plots show the expression status of MHCII and co‐signaling molecules CD40, CD80, B7H3, CD58, HVEM, PD‐L1, PD‐L2, and CD86 of S100 positive hrSCs in passage 1 (p1) and p2; technical replicates (same color); biological replicates (different colors). The histograms underneath depict one representative experiment (b, e, f, h, and j). Box plots represent the percentage of positive cells based on gates set in relation to unstained controls as displayed in the histograms (c, d, g, and i). Boxplots represent the mean fluorescence intensity (MFI). Boxes contain 50% of data and whiskers the upper and lower 25%; means are displayed as black horizontal lines. Each biological replicate is conducted with hrSCs isolated from a different donor nerve (b‐j). A two‐way ANOVA using a post‐hoc Holm p‐value correction was performed. *p ≤ 0.05; **p ≤ 0.01; and ***p ≤ 0.001

APCs modulate T‐cell activation through MHCII and the expression of co‐signaling molecules. Hence, we further investigated which co‐signaling molecules can be found on hrSCs. Therefore, we cultured hrSCs from eight different donor nerves and used flow cytometry to profile the expression of MHCII and selected co‐signaling molecules. As the interaction of SCs with immune cells has been described as a dynamic process (Gold et al., 1996), the analysis was performed at two time points, in passage one and passage two hrSC cultures. SC identity was determined by S100 expression, a well‐established SC marker, and showed that the mean purity of hrSCs cultures was 82% in passage one (p1) and 70% in passage 2 (p2) (Figure 2A). The S100 negative cells, presumably nerve‐associated fibroblasts, were excluded from further analysis (Figure 2A). In the S100 positive hrSCs, we quantified the surface expression of MHCII and co‐signaling molecules CD40, CD80, CD86, B7‐H3, HVEM, PD‐L1, PD‐L2, and CD58. About 76% and 87% of hrSCs were positive for MHCII in p1 and p2, respectively (Figure 2B), which is in line with our previously published observation that MHCII expression of hrSCs increased with prolonged culture time (Weiss et al., 2016). Further, p1 as well as p2 hrSCs demonstrated expression of CD40, CD80, B7‐H3, CD58, and HVEM (Figure 2C‐G). In contrast, neither PD‐L1 nor PD‐L2 were detected in p1 or p2 hrSC (Figure 2H‐I). Interestingly, most of p1 hrSCs were negative for CD86, while it was significantly upregulated in p2 cells (Figure 2J). Hence, hSCs indeed express—next to MHCII—several canonical co‐signaling molecules that are required for T‐cell activation and inhibition.

### 3.4 The stimulation of TLR3 and TLR4 had no effect on the expression of co‐signaling molecules in human repair‐related Schwann cells

**FIGURE 3:**
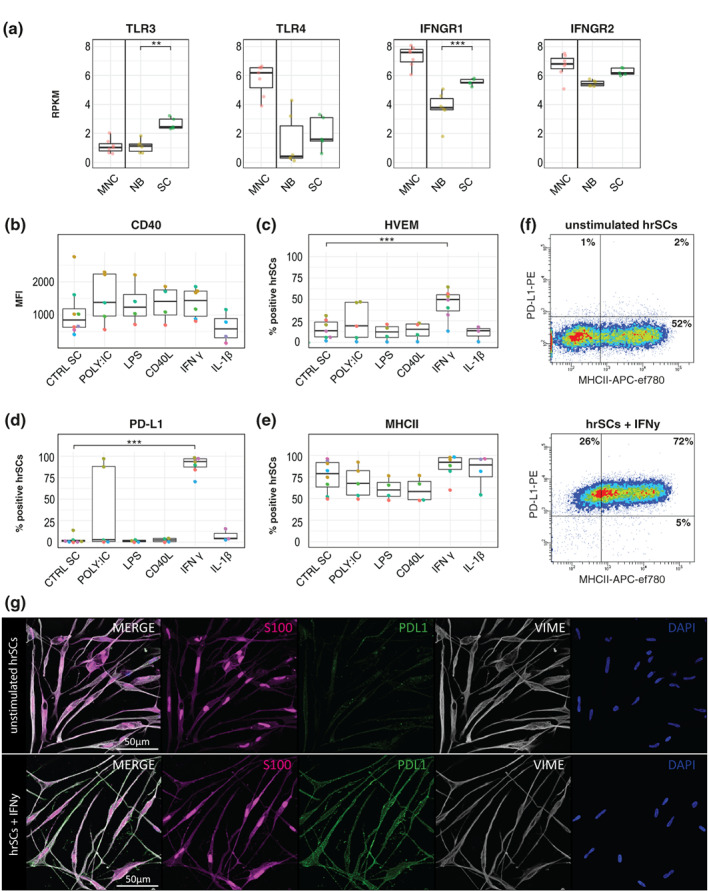
Immunophenotyping of human repair‐related Schwann cells upon toll‐like receptor and cytokine stimulation. (a) Enrichment of toll‐like receptor (TLR)‐related pathways. GSEA of RNA‐seq data sets of hrSCs compared to NB cultures. Box plots show the mRNA expression in reads per kilobase million (RPKM) of TLR3, IFNGR1, and IFNGR2 by RNA‐seq in NB cultures (NB, *n* = 5) and hrSCs (SC, *n* = 5). Bone marrow mononuclear cells (MNC, *n* = 5) are shown as reference (B‐E). FACS analysis of p1 cultures of hrSCs stimulated with POLY:IC, LPS, CD40L, IFNγ, and IL‐1β for 24 h. Box plots show the MFI of CD40 (B), percentage of HVEM (C), PD‐L1 (D), and MHCII (E) positive hrSCs in p1 cultures stimulated with POLY:IC, LPS, CD40L, IFNγ, and IL‐1β. Each biological replicate is conducted with hrSCs isolated from a different donor nerve. Boxes contain 50% of data and whiskers the upper and lower 25%. Means are displayed as black horizontal lines. All experiments were performed in at least four independent biological replicates. A two‐way ANOVA using a post‐hoc Holm *p*‐value correction was performed; *p ≤ 0.05; **p ≤ 0.01; and ***p ≤ 0.001. (f) Representative FACS plots showing PD‐L1 versus MHCII expression of p1 hrSCs either unstimulated (upper plot) and after IFNy stimulation. (g) Immunofluorescence image of p1 hrSCs at day 2 after purification without (upper panels) or with (lower panels) IFNy stimulation. HrSC cultures are stained for S100 (magenta), PD‐L1 (green), vimentin (gray), and DAPI (blue)

Professional as well as non‐professional APCs can upregulate the expression of co‐signaling molecules upon toll‐like receptor (TLR) ligation (Chen & Flies, 2013; Fitzgerald & Kagan, 2020). Our RNA‐seq data of hrSCs showed enrichment in TLR signaling in comparison to neuronal cells (Supplementary Table 4), which motivated us to explore the expression of co‐signaling molecules in response to inflammatory stimulation. Firstly, we investigated which TLRs are expressed by hrSCs to choose the corresponding ligands for further analysis. We found elevated expression levels of TLR1, TLR3, TLR4, and TLR6 mRNA (Figure 3A, Supplmentary Figure 1). As TLR1 and TLR6 mainly function as heterodimers with TLR2, which was not expressed, we focused on TLR3 and TLR4. We thus stimulated p1 hrSC with the TLR4 agonist LPS and TLR3 agonist POLY:IC for 24 h at concentrations known to activate APCs (de Oliveira et al., 2016) and subsequently analyzed the expression status of co‐signaling molecules (Figure 3B‐E, Supplmentary Figure 2). Interestingly, neither the addition of LPS nor POLY:IC caused a significant expressional change of the analyzed co‐signaling molecules in hrSCs (Figure 3B–E), which might be due to the low expression levels of TLR3 and TLR4 (Figure 3A). Upon stimulation with POLY:IC, a trend toward upregulation of CD40 and HVEM was seen, but substantial donor variance was observed (Figure 3B–E). CD40 is not only a co‐stimulatory molecule, but also a molecule that facilitates the activation of APCs upon binding of CD40‐ligand (Chen & Flies, 2013). CD40‐ligand however, did not affect the expression of CD40 or any other co‐signaling molecules tested (Figure 3B). Together these data show that TLR and CD40 ligation do not affect the expression of co‐signaling molecules.

### 3.5 Human repair‐related Schwann cells upregulate HVEM and PD‐L1 upon stimulation with IFNγ

Inflammatory processes in peripheral nerve tissues as well as upon injury responses involve the release of pro‐inflammatory mediators such as IFNγ and IL‐1β by macrophages (Chiu et al., 2012; Yao et al., 2010). Notably, we found that the expression of both IFNγ receptor genes, *IFNGR1* and *IFNGR2*, was upregulated in hrSC in comparison to neuronal cells (Figure 3A). We further investigated the response of hrSCs to IFNy as well as IL‐1β. While IL‐1β did not alter the expression of MHCII and any of the co‐stimulatory molecules tested, IFNy led to a significant increase in HVEM expression (Figure 3C). In addition, IFNγ stimulation induced a profound upregulation of PD‐L1 protein on the cell surface, which we found previously up‐regulated by hrSCs in vitro and ex vivo nerve explants at the transcript level (Figure 3D‐F, (Weiss et al., 2016)). PD‐L1 expression in response to IFNy was further validated on stimulated hrSC using multicolor immunofluorescence stainings for S100, PD‐L1 and vimentin (Figure 3G). Concordant with our flow cytometry data (Figure 3D‐F), unstimulated p1 hrSC did not show a notable PD‐L1 staining, whereas IFNγ stimulation strongly induced PD‐L1 protein expression (Figure 3G). Hence, the PD‐L1 mRNA is present in hrSCs but the protein is only expressed on the surface upon INFy stimulation. These findings show that hrSCs can respond to IFNy, but not to IL‐1β, by a significant up‐regulation of the co‐stimulatory molecule HVEM and the immune check‐point molecule PD‐L1.

### 3.6 Secretome analysis of human repair‐related Schwann cells reveals a broad spectrum of immunoactive mediators

**FIGURE 4:**
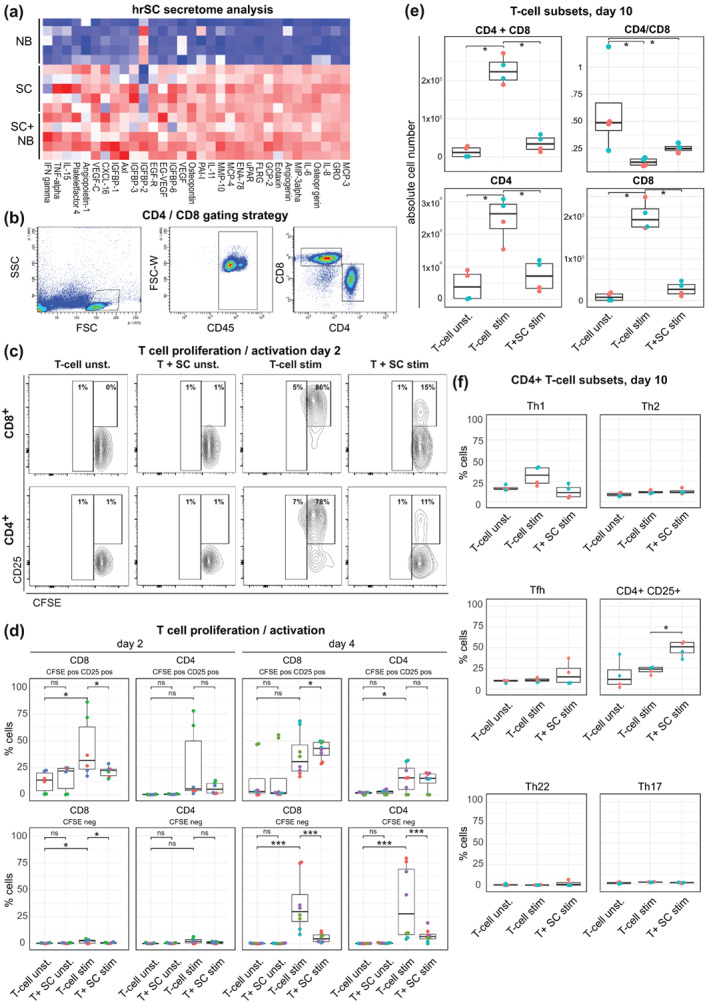
HrSCs secrete immunoactive mediators and inhibit allogenic T‐cell activation. (a) Secretome analysis by antibody array. Heatmap displays the top 32 differentially secreted proteins (*q* < 0.05, ∣log2FC >0.3∣) of hrSCs (*n* = 5) and SC‐NB co‐cultures (*n* = 5) versus NB cultures (as neuronal cell model) (*n* = 5). (b–f) allogeneic CD3^+^ T‐cells were cultured for 2, 4, or 10 days in the absence (Tcell) or presence of hrSCs (SC) and stimulated with anti‐CD3/CD28 beads and analyzed by flow cytometry. As control unstimulated T‐cells (Tcell unst.) were cultured. (b) Representative FACS plots show the gating strategy for CD4^+^ and CD8^+^ T‐cells. (c) Representative FACS plots showing CD25 expression against CFSE of T‐cells at day 2 of co‐cultivation and in control cultures. (d) Boxplots show the absolute number of CFSE^+^/CD25^+^ and CFSE^−^ CD4^+^ and CD8^+^ T‐cells at day 2 and day 4 based on gates set as illustrated in (b and c). (e) Boxplots show the absolute number of alive CD4^+^, CD8^+^ or combined CD4^+^and CD8^+^ cells, or the ratio of CD4^+^ over CD8^+^ and (F) percentage of CD4^+^ subsets evaluated via flow cytometry at day 10 based on gates set as published by Mahnke et al. (2013). (d–f) Boxes contain 50% of data and whiskers the upper and lower 25% means are displayed as black horizontal lines. All experiments were performed in at least three independent biological replicates. A two‐way ANOVA using a post‐hoc Holm *p*‐value correction was performed; *p ≤ 0.05; **p ≤ 0.01; and ***p ≤ 0.001

As the inducible expression of co‐signaling molecules by hrSCs together with their well‐described function to recruit macrophages and neutrophils (Stratton et al., 2016; Tzekova et al., 2014) points towards their active involvement in shaping a local immune response upon nerve injury, we further investigated a panel of immunoregulatory molecules secreted by hrSCs. In order to model the in vivo situation following nerve injury, supernatants of hrSCs cultured in the absence or presence of neuronal cells and neuronal cell control cultures were analyzed. Secretome analysis was performed using a protein array able to detect 274 different secreted factors. A total of 84 secreted molecules were unique to hrSCs and one was only secreted by NB cells (*q* < 0.05). None of the 84 proteins was differentially secreted in the SC‐NB co‐culture model as compared to SCs alone. We therefore considered these secreted proteins to be derived from hrSCs and further compared those to secreted proteins obtained from NB cultures (Supplementary Tables 5 and 6) [Correction added on 29 August, 2022, after first online publication: Included citation for Supplementary Table 6]. HrSC‐secreted factors included interleukins IL‐6, IL‐11, IL‐15, TNFα, IFNγ, molecules involved in phagocyte attraction, and activation such as MCP‐3, MCP‐4, or CXCL‐16, molecules for neutrophil attraction and activation such as GRO, MIP3‐alpha, IL‐8 (CXCL‐8), or ENA‐78 (Figure 4A). Many of these molecules are acting directly on lymphocytes, as for example IL‐6 that stimulates the proliferation of antibody producing B‐lymphocytes or IL‐15 stimulating T‐and NK‐ cell proliferation (Figure 4A, Supplementary Table 6). Interestingly, hrSCs also secreted osteopontin, a molecule involved in multiple processes including the induction of IFNγ through NF‐κB activation (Icer & Gezmen‐Karadag, 2018; O'Regan & Berman, 2000; Serlin et al., 2006). These findings demonstrate the plethora of immunoactive mediators secreted by hrSCs and suggests autocrine activity as well as paracrine modulation of myeloid cells and lymphocytes.

### 3.7 Human repair‐related Schwann cells reduce proliferation of allogeneic T‐cells

Based on the overlapping features of hrSCs and APCs, that is, phagocytosis, the expression of MHCII, co‐signaling and immune checkpoint molecules, and their inducible upregulation and secretion of T‐cell modulatory molecules, we next asked whether hrSCs might affect T‐cell activation and fate in the nerve injury context. Thus, we performed co‐culture assays of hrSCs and allogenic T‐cells. We used T‐cells from healthy donors stimulated with anti‐CD3/CD28 beads to simulate an inflammatory environment similarly to peripheral nerve injury in comparison to unstimulated T‐cells. In parallel, we compared this to T‐cell co‐cultures with human peripheral blood monocytes, which can be regarded as bona fide APCs. We then evaluated the impact of co‐culture assessing the total number of activated (CFSE^+^CD25^+^) and proliferated (CFSE^−/dim^) CD4^+^ and CD8^+^ T‐cells (Figure 4B‐C). In the absence of exogenous CD3/CD28 stimulation hrSCs were neither able to trigger T‐cell activation nor proliferation at days 2 or 4 (Figure 4C‐D). In contrast, monocytes were able to stimulate CD4^+^ and to a lesser extend CD8^+^ T‐cell activation and proliferation and this was even enhanced when CD3/CD28 stimulation was provided (Supplmentary Figure 3A‐B). Under the same conditions, however, the presence of hrSCs in co‐cultures significantly counteracted the effect of CD3/CD28 mediated increase in CFSE^+^CD25^+^ and proliferated (CFSE^−/dim^) CD8^+^ T‐cells (Figure 4D). At day 4, stimulation of T‐cells resulted in an increase of both, CD8^+^ and CD4^+^, CFSE^+^CD25^+^ as well as proliferated (CFSE^−/dim^) fraction. While proliferation was significantly reduced in CD8^+^ T‐cells by the presence of hrSCs in co‐culture, CFSE^+^CD25^+^ cells slightly increased (Figure 4D). A similar trend was observed in the CD4^+^ population (Figure 4D). This effect became even more apparent at day 10, when the total number of CD8^+^ as well as CD4^+^ T‐cells was reduced to numbers comparable to those of unstimulated T‐cells. (Figure 4E). As the previous investigations in immunodefficient (RAG1 knockout) mice suggest a time‐dependent role of especially CD4^+^ T‐cells in nerve injury (Serpe et al., 2003), we applied a FACS panel that interrogates the CD4^+^ T‐cell population for a potential shift among T‐helper subpopulations, i.e. Th1, Th2, Tfh, Th17, and Th22, using a 13‐plex flow cytometry panel (Mahnke et al., 2013). Notably, there was no clear trend toward a specific CD4^+^ Th subset, yet a significantly higher percentage of CD4^+^CD25^+^ cells was detected (Figure 4F). As CD8^+^ T‐cells were prevalent in these long‐term cultures (probably as a result of massive CD4 expansion, exhaustion and cell death in culture), we investigated PD‐1 as activation and exhaustion marker, CCR6, which is expressed by effector memory T‐cells, and CD25 expression also on the CD8^+^ T‐cell population. However, the fraction of CD25^+^ and CCR6^+^ CD8^+^ cells did not change significantly in SC‐co‐cultures compared to stimulated T‐cells alone. A decrease in PD‐1 expressing CD8^+^ cells was observed, which is in line with the reduced activation of CD8^+^ T‐cells in the presence of hrSCs (Supplmentary Figure 4). In summary, hrSCs delayed proliferation of CD8^+^ and CD4^+^ T‐cells, while promoting the long‐term survival or potential switch toward a CD4^+^CD25^+^ phenotype. This highlights the functional difference between monocytes as classical antigen‐presenting cells and hrSCs, which exerted an inhibitory function.

## 4 DISCUSSION

Building on our previous characterization of the transcriptome and proteome of human repair‐type SCs (Weiss et al., 2016) and studies suggesting an MHCII‐mediated immune response of human SCs in disease (Meyer zu Hörste et al., 2010; Weiss et al., 2021), we aimed to provide novel insight into the immunocompetence of human SCs in an injury condition. Using the primary human repair‐related SCs, hrSCs, as a unique model to study their immunophenotype and associated functional aspects, our study presents several layers of evidence that hrSCs possess features and functions of APCs and are capable of mediating T‐cell‐dependent immunity. We demonstrate that hrSCs can express CD40, CD80, B7H3, CD58, CD86, HVEM, and PD‐L1 in addition to MHCII, secrete numerous immunomodulatory molecules, and inhibit allogeneic T‐cell activation. It is well accepted that many functions of professional APCs, including the presentation of antigen via MHCII, the expression of co‐signaling molecules and the secretion of anti‐ and pro‐inflammatory molecules, are also carried out by non‐professional APCs such as mast cells, eosinophils, and non‐hematopoietic cells like epithelial cells (Kambayashi & Laufer, 2014; Schuijs et al., 2019). With this study, we add compelling evidence that hrSCs could also act as non‐professional APCs that may modulate the inflammatory processes within injured nerves. Studying the interaction of primary hrSCs and T‐cells allowed the development of a broadly applicable functional in vitro model that contributes to the ongoing research in the field of neuroinflammatory disorders, regenerative medicine, and immune oncology.

### 4.1 Human repair‐related SCs possess features of antigen‐presenting cells

We and others have previously shown that hrSCs are able to perform phagocytosis of cell extrinsic material along their processes and express MHCII on their surface (Weiss et al., 2016). In line with our transcriptome and proteome study on ex vivo degenerated human nerves (Weiss et al., 2016; Welleford et al., 2020). Single cell and spatial transcriptomics studies of human‐injured nerves will provide additional information about the expression dynamics of MHCII‐ and phagocytosis‐related genes in SCs during injury, but to date such information is not available.

In addition, this study demonstrates that hrSCs accumulate phagocytosed material in the cell body over time. Upon nerve injury, SCs are known to be highly phagocytic, degrade their own myelin by phagocytic and autophagic processes (Gomez‐Sanchez et al., 2015; Jang et al., 2016; Lutz et al., 2017). This specific cellular state was recently also described as “demyelinating SC phenotype” that holds specific properties required to clear the myelin sheath during Wallerian degeneration (Park et al., 2019). These demyelinating SCs might represent an earlier stage of repair SCs and express MHCII alongside with co‐inhibitory molecules to prevent auto‐immunity to myelin components.

It is well‐established that not only classical APCs but also non‐professional APCs, like mast cells or epithelial cells, are capable of phagocytosis and antigen presentation via MHC II to CD4^+^ T‐cells (Kambayashi & Laufer, 2014; Schuijs et al., 2019). As non‐professional APCs of non‐hematopoietic origin do not primarily migrate to lymph nodes, their role in priming naïve T‐cells may be less relevant, but their modulation of a local T‐cell responses is widely accepted (Kambayashi & Laufer, 2014). Thus, the expression of co‐signaling molecules alongside with MHCII and the secretion of other immunomodulatory molecules defines the effect of non‐professional APCs in different tissues and conditions. In this study, we show that hrSC are able to express the co‐signaling molecules CD58, CD80, and CD86 in addition to MHCII. These molecules are associated with an activation of T‐cells (Chen & Flies, 2013; Greenwald et al., 2004).

Interestingly, a study comparing nerve biopsies of healthy patients and patients with chronic inflammatory demyelinating polyneuropathy (CIDP) identified that CD58 expressing SCs were only found in the latter (Van Rhijn et al., 2000). This could be due to a similarity in the role of SCs after nerve injury and during the interaction with immune cells in autoimmune diseases. However, Van Rhijn et al. did not observe CD86 or CD80 expressing SCs in healthy or CIDP patients, which indicates that the expression of co‐signaling molecules detected in our in vitro model might reflect a unique feature of hrSCs. Of note, our primary human SCs were isolated from peripheral nerves obtained after amputational surgeries and knowledge on pre‐existing conditions such as non‐diagnosed neuropathies and medication are limited. However, we observed consistent and reproducible effects throughout our molecular, phenotypic and functional characterization of hrSCs.

In addition to surface expression of co‐signaling molecules, we found that hrSC secrete a variety of immunomodulators. This is in line with the previous studies on human SCs that demonstrated the secretion of IL‐6, IL‐8, IL‐15, and MCP‐1 (Ozaki et al., 2008; Rutkowski et al., 1999). Our study enriches the repertoire of secreted hrSC molecules by cytokines like IL‐11 and chemoattractants such as MCP‐3, MCP‐4, CXCL‐16, GRO, and MIP3α suggesting an unexpected functional diversity. In contrast to Rutkowski et al., 1999, we did not detect the expression of IL‐1β in our assay (Rutkowski et al., 1999). The secreted repertoire supports a predominant role of SC in attracting lymphocytes, monocytes and neutrophils after nerve injury. It will be of importance to delineate the function of each of these factors—especially in the context of interactions between hrSC, macrophages and T‐cells—to further investigate the time and context‐dependent role of hrSCs in modulating the immune response after nerve injury. Taken together, these findings demonstrate that hrSCs express the co‐signaling molecules CD58, CD80, and CD86 together with MHCII and provide novel insight into the repertoire of hrSC secreted molecules with immunoregulatory functions.

### 4.2 Inhibition of allogeneic T‐cell activation—evidence for an immuno‐regulatory function of human repair SCs

Furthermore, we show that the exposure to hrSCs causes a delayed or even abrogated CD4^+^ T‐cell proliferation and activation. In line with this finding, we demonstrate that SCs express co‐inhibitory molecules such as B7‐H3, HVEM and provide the first report that hrSC upregulate PD‐L1 after stimulation with IFNγ. As not only the activation, but also the termination and resolution of inflammation through surface expression of inhibitory molecules is a hallmark of APCs, the presence of these molecules in hrSCs is remarkable. The source for IFNγ release in inflammatory tissues are mainly NK, CD4^+^ and CD8^+^ T‐cells as well as macrophages. Human repair‐related SCs may even trigger the release of IFNy via secreted osteopontin that has been shown to induce IFNγ production in T‐cells (Ashkar et al., 2000; Icer & Gezmen‐Karadag, 2018; Serlin et al., 2006) and could potentially act in an autocrine manner to induce IFNy production in hrSCs. Indeed, in this study we show that hrSCs are also capable of IFNγ secretion. Whether the autocrine production of IFNγ by hrSCs or the paracrine IFNγ released by other cell types present at the site of nerve injury induces the surface expression of PD‐L1 on hrSCs remains to be determined.

We further observed that delayed T‐cell activation was not accompanied by a shift toward any particular T helper subset, but rather resulted in CD4^+^ T‐cells with high CD25 expression, which may represent a regulatory or exhausted phenotype. The hypothesis of regulatory/exhausted T‐cells is supported by previous observations in rodent models (Meyer zu Horste et al., 2014; Meyer zu Hörste et al., 2010; F.‐J. Wang et al., 2018; Wang et al., 2014). Similarly, it has been shown that non‐professional APCs like type II alveolar epithelial cells can prime antigen‐specific CD4^+^ T‐cells toward regulatory T‐cells (Kambayashi & Laufer, 2014). This suggests that hrSCs in their activated state might, despite high MHCII, CD40, CD80, and CD58 expression and secretion of pro‐inflammatory cytokines IL‐6, IL‐8, TNFα and IFNγ, adopt an antigen‐presenting cell phenotype similar to M2 macrophages, which tightly control and terminate T‐cell responses via B7‐H3 and the PD‐L1/PD‐1 axis. Thus, a balance between the initiation and termination of an immune response may be essentially controlled through repair SCs during the multistep process of nerve regeneration.

The expression of PD‐L1 by hrSCs after stimulation with IFNγ supports the idea of a time and situation‐dependent role of repair SCs. It is tempting to speculate that repair SCs initiate the termination of the inflammatory response they helped to induce as first responders to nerve injury. Our data suggest that repair SCs might possess an immunoregulatory function that could prevent unnecessary damage to the neuronal environment by terminating an exceeding immune response, in which large amounts of IFNγ are produced by immune cells recruited to the site of injury. To address this possibility, deeper phenotypic and functional characterization of especially CD4^+^CD25^+^ T‐cells in vitro*/*ex vivo will be required in the future.

Thus, manipulating the balance of pro‐ and anti‐inflammatory profile of repair SCs might represent a novel therapeutic target in regenerative and pathological processes.

A potential limitation of our in vitro model is the use of beads coated with CD3 and CD28 as source of exogenous T‐cell stimulation. While we cannot exclude that beads might be phagocytosed by hrSCs or monocytes in culture and reduce the availability of beads to stimulate T‐cells, two factors indicate the validity of our results. Firstly, signaling via TCR engagement is a rapid process leading to down‐stream phosphorylation of ZAP70 within minutes, whereas uptake of 1 um latex beads by hrSCs was only accomplished within hours after exposure. After 2 h, single hrSCs showed uptake of beads, while after 16 h, a major fraction of hrSCs contained latex beads (this study and (Weiss et al., 2016)). Secondly, monocytes—classical antigen‐presenting cells—and CD3/CD28 activator beads, showed an additive effect in their T‐cell stimulatory capacity, indicating that indeed T‐cells are activated by CD3/CD28 beads even in the presence of phagocytes.

### 4.3 Impications for the field of immuno oncology

Importantly, it could be shown that SCs play a fundamental role in certain immune‐oncological processes. SCs with a repair‐related phenotype (including MHCII expression) are attracted by favorable forms of peripheral neuroblastic tumors, neuroblastomas, of a genetic subtype and trigger tumor cell maturation/differentiation and apoptosis, a phenomenon which could also be recapitulated in in vitro experiments (Weiss et al., 2021). These tumors, in comparison to their malignant counterpart, frequently show prominent MHCII+ and CD3+ immune cell infiltrates (Ambros et al., 1996; Weiss et al., 2021). It will be interesting to study the composition of these infiltrates and whether and how SCs contribute to their recruitment and modulation. [Correction added on 29 August, 2022, after first online publication: Included section 4.3].

## 5 CONCLUSION

In summary, we here provide in vitro evidence that human SCs in an injury condition adopt functions of APCs, that is, phagocytosis, up‐regulation of MHCII and co‐signaling molecules, secretion of an array of immunoregulatory molecules, and repression of T‐cell activation. Our data suggest that repair SCs can participate in the termination of the inflammatory response to prevent excessive tissue damage and allow nerve regeneration. The molecules expressed and secreted by hrSC presented in this study will help to understand their complex interplay with immune cells after injury and has implications for the field of neuro‐inflammatory disorders, regenerative medicine, and immune oncology.

## AUTHOR CONTRIBUTIONS

Sabine Taschner‐Mandl conceptualized the project; Jakob Berner, Tamara Weiss and Sabine Taschner‐Mandl planned experiments, performed research, analyzed and interpreted data and wrote the manuscript; Helena Sorger and Fikret Rifatbegovic performed research and analyzed data; Max Kauer developed bioinformatics tools and analyzed data; Alexander Dohnal, Kaan Boztug and Peter Steinberger provided essential reagents, planned experiments and interpreted data; Reinhard Windhager provided essential material; Peter F. Ambros and Inge M. Ambros interpreted data; all authors reviewed the manuscript.

## CONFLICT OF INTEREST

The authors declare no conflict of interest.

## Supporting information


**Supplementary Table 1.** List of antibodies.
**Supplementary Table 2.** List of differentially expressed genes hrSC vs NB cells
**Supplementary Table 3.** Go Term analysis of differentially expressed genes hrSC vs NB cells
**Supplementary Table 4.** Gene set enrichment analysis hrSCs vs NB cells
**Supplementary Table 5.** Proteinarray data dat

**Supplementary Figure 1** mRNA expression of Toll‐like receptors, interferon receptors and co‐signaling molecules. Boxplots show mRNA levels (RPKM) in hrSCs (*n* = 5) versus NB cells (*n* = 5). MNCs are shown as reference. (A) Toll‐like receptors (B) interferon receptors and (C) co‐stimulatory and inhibitory molecules. Boxes contain 50% of data and whiskers the upper and lower 25% means are displayed as black horizontal lines.

**Supplementary Figure 2** Flow cytometry‐based phenotyping of MHCII and co‐signaling molecules upon inflammatory stimulation. Box plots show the expression status of CD58 (A), PDL2 (B), CD80 (C), CD83 (D), CD86 (E) and B7H3 (F) of S100 positive hrSCs after stimulation with POLY:IC, LPS, CD40L, IFNγ and IL‐1β; *n* = 9; technical replicates (same color); biological replicates (different color). Each biological replicate is conducted with hrSCs isolated from a different donor nerve. (A, B, E, and F) Boxplots represent the percentage of positive cells based on gates set in relation to unstained controls. (C, D) Boxplots represent the mean fluorescence intensity (MFI). Boxes contain 50% of data and whiskers the upper and lower 25%; means are displayed as black horizontal lines. A two‐way ANOVA using a post‐hoc Holm *p*‐value correction was performed; *p ≤ 0.05; **p ≤ 0.01; and ***p ≤ 0.001.

**Supplementary Figure 3** T‐cell activation and proliferation in co‐cultures with human peripheral blood monocytes. Co‐cultures were performed in the absence or presence of CD3/CD28 activation. (A) Representative FACS plots showing CD25 expression against CFSE of T‐cells at day 2 of co‐cultivation and in control cultures. (B) Boxplots show the CFSE^+^/CD25^+^ and CFSE^−^ CD4^+^ and CD8^+^ T‐cells at day 2 and day 4 based on gates set as illustrated in (A). Boxplots represent the percentage of positive cells based on the parental population. Boxes contain 50% of data and whiskers the upper and lower 25%; means are displayed as black horizontal lines. A two‐way ANOVA using a post‐hoc Holm *p*‐value correction was performed; *p ≤ 0.05; **p ≤ 0.01; and ***p ≤ 0.001.

**Supplementary Figure 4** CD8 T‐cell subpopulations at day 10 after co‐cultivation with hrSCs and stimulation with CD3/28 beads (A) Representative FACS plots showing the gating strategy for CD25 positive, CCR6 positive and PD1 positive T‐cells after gating for CD8 positive T‐cells as illustrated in Figure 4. (B) Boxplots represent the percentage of positive cells based on the parental population. Boxes contain 50% of data and whiskers the upper and lower 25%; means are displayed as black horizontal lines. A two‐way ANOVA using a post‐hoc Holm *p*‐value correction was performed; *p ≤ 0.05; **p ≤ 0.01; and ***p ≤ 0.001.

## Data Availability

Data and code availability; no original code has been generated in this study. Original/source data for all figures and supplementary figures are available upon request. RNA‐sequencing data are available at the Gene Expression Omnibus (GEO) repository (Home ‐ GEO ‐ NCBI (nih.gov) under the identifier GSE94A035, GSE90711 and GSE90711.
