# Supplementary material for: Human repair‐related Schwann cells adopt functions of antigen‐presenting cells in vitro
Source: Glia. 2022 Aug 17;70(12):2361–77. doi: 10.1002/glia.24257 (PMC9804420; doi:10.1002/glia.24257)
Supplement: Supplementary file 1 — Supplementary Table 1. List of antibodies. Supplementary Table 2. List of differentially expressed genes hrSC vs NB cells Supplementary Table 3. Go Term analysis of differentially expressed genes hrSC vs NB cells Supplementary Table 4. Gene set enrichment analysis hrSCs vs NB cells Supplementary Table 5. Proteinarray data dat [file GLIA-70-2361-s004.zip › glia24257-sup-0006-Table6.docx]

Supplementary Table 6

| Proteins | Alternative names | Function | Referrences |
| --- | --- | --- | --- |
| MCP3 | CCL7; | attraction of macrophages and lymphocytes | (Cheng et al., 2014) |
| CXCL1 | GROa; GRO1; NAP3 | attraction and activation of neutrophils | (Korbecki, Barczak, Gutowska, Chlubek, & Baranowska-Bosiacka, 2022; Korbecki, Gąssowska-Dobrowolska, et al., 2022) |
| IL-8 | CXCL8 | attraction of leukocytes, neutrophils, induces angiogenesis | (Mukaida, Harada, & Matsushima, 1998; Teijeira et al., 2021) |
| Osteoprotegerin | OPG, OCIF, TNFRSF11B | decoy receptor for RANKL, negative regulation of bone resorption | (Boyce & Xing, 2007; Feige, 2001) |
| MIP3a | CCL20; LARC; | attraction of lymphoytes, repression of myeloid progenitor proliferation | (Kondo, Takata, & Takiguchi, 2007; A. Y. S. Lee & Körner, 2019) |
| Angiogenin | ANG; RAA1 | induces angiogenesis | (Sarangdhar & Allam, 2021) |
| Eotaxin | CCL11; SCYA11 | attraction of eosinophils | (Williams, 2015) |
| GCP-2 | CXCL6; SCYB6 | attraction of neutrophils, direct antibacterial action | (Linge et al., 2008) |
| FLRG | FSTL3;FSRP | inhibits osteoclast formation, involved in hematopoietic progenitor differentiation | (Maguer-Satta & Rimokh, 2004) |
| uPAR | PLAUR; URKR | plasmin activator on cell surface | (Montuori & Ragno, 2014; Smith & Marshall, 2010) |
| MCP4 | CCL13 | attraction of monocytes, lymphocytes, eosinophils and basophils | (Mendez-Enriquez & García-Zepeda, 2013) |
| MMP-10 |  | breaks down extracellular matrix | (Rodriguez et al., 2008) |
| IL-11 | AGIF | stimulates T-cell dependent development of B-cells, production of platelets | (Fung et al., 2022) |
| PAI-1 | SERPIN1; PAI | involved in innate antiviral immunity and fibrinolysis | (Dellas & Loskutoff, 2005) |
| Osteopontin | SPP1; ETA1; | cytokine that upregulates expression of IFN and IL12 | (Ashkar et al., 2000; Icer & Gezmen-Karadag, 2018; O’Regan & Berman, 2000; Serlin et al., 2006) |
| VEGF | VEGF | angiogenesis | (Shaik-Dasthagirisaheb et al., 2013) |
| IGFBP-6 | IBP-6 | mediation of cell growth and metabolism | (Liso et al., 2022) |
| EG-VEGF | PROK1; PRK1 | angiogenesis | (Alfaidy et al., 2014) |
| IGFBP-2 | IBP-2 | Mediation of cell growth and metabolism | (Li et al., 2020) |
| IGFBP-3 | IBP-3 | Mediation of cell growth and metabolism | (Ranke, 2015) |
| Axl | UFO; JTK11; Tyro7 | binding of GAS6 mediating innate immune response (a growth factor for SCs shown to be produced by macrophages upon injury | (Lutz et al., 2017; Zhu, Wei, & Wei, 2019) |
| IGFBP-1 | PP12; IBP1 | Mediation of cell growth and metabolism | (Hoeflich & Russo, 2015) |
| CXCL16 |  | positive regulator of cell growth; response to IFNγ and TNF | (Korbecki et al., 2021) |
| VEGFC | VRP | angiogenesis | (Olofsson, Jeltsch, Eriksson, & Alitalo, 1999) |
| Angiopoetin-1 | ANGPT1; ANG1 | angiogenesis | (Skóra et al., 2021) |
| Plateletfactor4 | PF4; CXCL4 | chemokine attracts numerous cell types and acts as a negative regulator of hematopoiesis, angiogenesis and T-cell function | (Kowalska, Rauova, & Poncz, 2010) |
| IL-15 |  | regulates T and NK cell activation and proliferation, survival and proliferation of memory CD8+ cells | (Rentzos & Rombos, 2012; S. Zhang, Zhao, Bai, Handley, & Shan, 2021) |
| TNFa | TNF | Pro-inflammatory cytokine, Pyrogen, induces IL-1 secretion; endothelial cell activation; Neutrophil Activation, induction of acute phase proteins | (Gough & Myles, 2020) |
| INFg |  | Macrophage activation; B cell isotype switching ;Th1 differentiation; increased antigen processing and presentation to T cells | (Schoenborn & Wilson, 2007) |

**Supplementary Table 6 │ functions of hrSC secreted factors.** Differential protein secretion of hrSCs versus NB cell lines determined by protein array.

References

Alfaidy, N., Hoffmann, P., Boufettal, H., Samouh, N., Aboussaouira, T., Benharouga, M., … Brouillet, S. (2014). The multiple roles of EG-VEGF/PROK1 in normal and pathological placental angiogenesis. *BioMed Research International*, *2014*, 451906. https://doi.org/10.1155/2014/451906

Ashkar, S., Weber, G. F., Panoutsakopoulou, V., Sanchirico, M. E., Jansson, M., Zawaideh, S., … Cantor, H. (2000). Eta-1 (Osteopontin): An Early Component of Type-1 (Cell-Mediated) Immunity. *Science*, *287*(5454), 860–864. https://doi.org/10.1126/science.287.5454.860

Boyce, B. F., & Xing, L. (2007). Biology of RANK, RANKL, and osteoprotegerin. *Arthritis Research & Therapy*, *9 Suppl 1*(Suppl 1), S1. https://doi.org/10.1186/ar2165

Cheng, J. W., Sadeghi, Z., Levine, A. D., Penn, M. S., von Recum, H. A., Caplan, A. I., & Hijaz, A. (2014). The role of CXCL12 and CCL7 chemokines in immune regulation, embryonic development, and tissue regeneration. *Cytokine*, *69*(2), 277–283. https://doi.org/10.1016/j.cyto.2014.06.007

Dellas, C., & Loskutoff, D. J. (2005). Historical analysis of PAI-1 from its discovery to its potential role in cell motility and disease. *Thrombosis and Haemostasis*, *93*(4), 631–640. https://doi.org/10.1160/TH05-01-0033

Feige, U. (2001). Osteoprotegerin. *Annals of the Rheumatic Diseases*, *60 Suppl 3*(Suppl 3), iii81-4. https://doi.org/10.1136/ard.60.90003.iii81

Fung, K. Y., Louis, C., Metcalfe, R. D., Kosasih, C. C., Wicks, I. P., Griffin, M. D. W., & Putoczki, T. L. (2022). Emerging roles for IL-11 in inflammatory diseases. *Cytokine*, *149*, 155750. https://doi.org/10.1016/j.cyto.2021.155750

Gough, P., & Myles, I. A. (2020). Tumor Necrosis Factor Receptors: Pleiotropic Signaling Complexes and Their Differential Effects. *Frontiers in Immunology*, *11*, 585880. https://doi.org/10.3389/fimmu.2020.585880

Hoeflich, A., & Russo, V. C. (2015). Physiology and pathophysiology of IGFBP-1 and IGFBP-2 - consensus and dissent on metabolic control and malignant potential. *Best Practice & Research. Clinical Endocrinology & Metabolism*, *29*(5), 685–700. https://doi.org/10.1016/j.beem.2015.07.002

Icer, M. A., & Gezmen-Karadag, M. (2018). The multiple functions and mechanisms of osteopontin. *Clinical Biochemistry*, *59*, 17–24. https://doi.org/10.1016/j.clinbiochem.2018.07.003

Kondo, T., Takata, H., & Takiguchi, M. (2007). Functional expression of chemokine receptor CCR6 on human effector memory CD8+ T cells. *European Journal of Immunology*, *37*(1), 54–65. https://doi.org/10.1002/eji.200636251

Korbecki, J., Bajdak-Rusinek, K., Kupnicka, P., Kapczuk, P., Simińska, D., Chlubek, D., & Baranowska-Bosiacka, I. (2021). The Role of CXCL16 in the Pathogenesis of Cancer and Other Diseases. *International Journal of Molecular Sciences*, *22*(7). https://doi.org/10.3390/ijms22073490

Korbecki, J., Barczak, K., Gutowska, I., Chlubek, D., & Baranowska-Bosiacka, I. (2022). CXCL1: Gene, Promoter, Regulation of Expression, mRNA Stability, Regulation of Activity in the Intercellular Space. *International Journal of Molecular Sciences*, *23*(2). https://doi.org/10.3390/ijms23020792

Korbecki, J., Gąssowska-Dobrowolska, M., Wójcik, J., Szatkowska, I., Barczak, K., Chlubek, M., & Baranowska-Bosiacka, I. (2022). The Importance of CXCL1 in Physiology and Noncancerous Diseases of Bone, Bone Marrow, Muscle and the Nervous System. *International Journal of Molecular Sciences*, *23*(8). https://doi.org/10.3390/ijms23084205

Kowalska, M. A., Rauova, L., & Poncz, M. (2010). Role of the platelet chemokine platelet factor 4 (PF4) in hemostasis and thrombosis. *Thrombosis Research*, *125*(4), 292–296. https://doi.org/10.1016/j.thromres.2009.11.023

Lee, A. Y. S., & Körner, H. (2019). The CCR6-CCL20 axis in humoral immunity and T-B cell immunobiology. *Immunobiology*, *224*(3), 449–454. https://doi.org/10.1016/j.imbio.2019.01.005

Li, T., Forbes, M. E., Fuller, G. N., Li, J., Yang, X., & Zhang, W. (2020). IGFBP2: integrative hub of developmental and oncogenic signaling network. *Oncogene*, *39*(11), 2243–2257. https://doi.org/10.1038/s41388-020-1154-2

Linge, H. M., Collin, M., Nordenfelt, P., Mörgelin, M., Malmsten, M., & Egesten, A. (2008). The human CXC chemokine granulocyte chemotactic protein 2 (GCP-2)/CXCL6 possesses membrane-disrupting properties and is antibacterial. *Antimicrobial Agents and Chemotherapy*, *52*(7), 2599–2607. https://doi.org/10.1128/AAC.00028-08

Liso, A., Venuto, S., Coda, A. R. D., Giallongo, C., Palumbo, G. A., & Tibullo, D. (2022). IGFBP-6: At the Crossroads of Immunity, Tissue Repair and Fibrosis. *International Journal of Molecular Sciences*, *23*(8). https://doi.org/10.3390/ijms23084358

Lutz, A. B., Chung, W.-S., Sloan, S. A., Carson, G. A., Zhou, L., Lovelett, E., … Barres, B. A. (2017). Schwann cells use TAM receptor-mediated phagocytosis in addition to autophagy to clear myelin in a mouse model of nerve injury. *Proceedings of the National Academy of Sciences*, *114*(38), E8072–E8080. https://doi.org/10.1073/pnas.1710566114

Maguer-Satta, V., & Rimokh, R. (2004). FLRG, member of the follistatin family, a new player in hematopoiesis. *Molecular and Cellular Endocrinology*, *225*(1–2), 109–118. https://doi.org/10.1016/j.mce.2004.07.009

Mendez-Enriquez, E., & García-Zepeda, E. A. (2013). The multiple faces of CCL13 in immunity and inflammation. *Inflammopharmacology*, *21*(6), 397–406. https://doi.org/10.1007/s10787-013-0177-5

Montuori, N., & Ragno, P. (2014). Role of uPA/uPAR in the modulation of angiogenesis. *Chemical Immunology and Allergy*, *99*, 105–122. https://doi.org/10.1159/000353310

Mukaida, N., Harada, A., & Matsushima, K. (1998). Interleukin-8 (IL-8) and monocyte chemotactic and activating factor (MCAF/MCP-1), chemokines essentially involved in inflammatory and immune reactions. *Cytokine & Growth Factor Reviews*, *9*(1), 9–23. https://doi.org/10.1016/s1359-6101(97)00022-1

O’Regan, A., & Berman, J. S. (2000). Osteopontin: a key cytokine in cell-mediated and granulomatous inflammation. *International Journal of Experimental Pathology*, *81*(6), 373–390. https://doi.org/10.1046/j.1365-2613.2000.00163.x

Olofsson, B., Jeltsch, M., Eriksson, U., & Alitalo, K. (1999). Current biology of VEGF-B and VEGF-C. *Current Opinion in Biotechnology*, *10*(6), 528–535. https://doi.org/10.1016/s0958-1669(99)00024-5

Ranke, M. B. (2015). Insulin-like growth factor binding-protein-3 (IGFBP-3). *Best Practice & Research. Clinical Endocrinology & Metabolism*, *29*(5), 701–711. https://doi.org/10.1016/j.beem.2015.06.003

Rentzos, M., & Rombos, A. (2012). The role of IL-15 in central nervous system disorders. *Acta Neurologica Scandinavica*, *125*(2), 77–82. https://doi.org/10.1111/j.1600-0404.2011.01524.x

Rodriguez, J. A., Orbe, J., Martinez de Lizarrondo, S., Calvayrac, O., Rodriguez, C., Martinez-Gonzalez, J., & Paramo, J. A. (2008). Metalloproteinases and atherothrombosis: MMP-10 mediates vascular remodeling promoted by inflammatory stimuli. *Frontiers in Bioscience : A Journal and Virtual Library*, *13*, 2916–2921. https://doi.org/10.2741/2896

Sarangdhar, M. A., & Allam, R. (2021). Angiogenin (ANG)-Ribonuclease Inhibitor (RNH1) System in Protein Synthesis and Disease. *International Journal of Molecular Sciences*, *22*(3). https://doi.org/10.3390/ijms22031287

Schoenborn, J. R., & Wilson, C. B. (2007). Regulation of Interferon‐γ During Innate and Adaptive Immune Responses (Vol. 96, pp. 41–101). Academic Press. https://doi.org/https://doi.org/10.1016/S0065-2776(07)96002-2

Serlin, D. M., Kuang, P. P., Subramanian, M., O’Regan, A., Li, X., Berman, J. S., & Goldstein, R. H. (2006). Interleukin-1beta induces osteopontin expression in pulmonary fibroblasts. *Journal of Cellular Biochemistry*, *97*(3), 519–529. https://doi.org/10.1002/jcb.20661

Shaik-Dasthagirisaheb, Y. B., Varvara, G., Murmura, G., Saggini, A., Potalivo, G., Caraffa, A., … Pandolfi, F. (2013). Vascular endothelial growth factor (VEGF), mast cells and inflammation. *International Journal of Immunopathology and Pharmacology*. England. https://doi.org/10.1177/039463201302600206

Skóra, J. P., Antkiewicz, M., Kupczyńska, D., Kulikowska, K., Strzelec, B., Janczak, D., & Barć, P. (2021). Local intramuscular administration of ANG1 and VEGF genes using plasmid vectors mobilizes CD34+ cells to peripheral tissues and promotes angiogenesis in an animal model. *Biomedicine & Pharmacotherapy = Biomedecine & Pharmacotherapie*, *143*, 112186. https://doi.org/10.1016/j.biopha.2021.112186

Smith, H. W., & Marshall, C. J. (2010). Regulation of cell signalling by uPAR. *Nature Reviews. Molecular Cell Biology*, *11*(1), 23–36. https://doi.org/10.1038/nrm2821

Teijeira, A., Garasa, S., Ochoa, M. C., Villalba, M., Olivera, I., Cirella, A., … Melero, I. (2021). IL8, Neutrophils, and NETs in a Collusion against Cancer Immunity and Immunotherapy. *Clinical Cancer Research : An Official Journal of the American Association for Cancer Research*, *27*(9), 2383–2393. https://doi.org/10.1158/1078-0432.CCR-20-1319

Williams, T. J. (2015). Eotaxin-1 (CCL11). *Frontiers in Immunology*, *6*, 84. https://doi.org/10.3389/fimmu.2015.00084

Zhang, S., Zhao, J., Bai, X., Handley, M., & Shan, F. (2021). Biological effects of IL-15 on immune cells and its potential for the treatment of cancer. *International Immunopharmacology*, *91*, 107318. https://doi.org/10.1016/j.intimp.2020.107318

Zhu, C., Wei, Y., & Wei, X. (2019). AXL receptor tyrosine kinase as a promising anti-cancer approach: functions, molecular mechanisms and clinical applications. *Molecular Cancer*, *18*(1), 153. https://doi.org/10.1186/s12943-019-1090-3
