# Supplementary material for: Human repair‐related Schwann cells adopt functions of antigen‐presenting cells in vitro
Source: Glia. 2022 Aug 17;70(12):2361–77. doi: 10.1002/glia.24257 (PMC9804420; doi:10.1002/glia.24257)
Supplement: Supplementary file 1 — Supplementary Table 1. List of antibodies. Supplementary Table 2. List of differentially expressed genes hrSC vs NB cells Supplementary Table 3. Go Term analysis of differentially expressed genes hrSC vs NB cells Supplementary Table 4. Gene set enrichment analysis hrSCs vs NB cells Supplementary Table 5. Proteinarray data dat [file GLIA-70-2361-s004.zip › glia24257-sup-0001-Table1.docx]

Supplementary Table 1

| **1^st^ antibodies** | | | | **Application** | | |
| --- | --- | --- | --- | --- | --- | --- |
| Antigen | Species | catalog No | company | dilution | comment | |
| S100 | rabbit | #Z0311 | DAKO | 1:200 | 1 hr, RT, perm | |
| vimentin | chicken | #AB5733 | Merck Millipore | 1:200 | 1 hr, RT, perm | |
| NGFR | rabbit | #8238S | CellSignaling | 1:300 | o.n., 4°C | |
| PD-L1 | Mouse | #4274539 | eBioscience | 1:50 | o.n. 4°C | |
| HLA-DR-α1 | mouse | #M0746 | DAKO | 1:50 | o.n., 4°C | |
|  | | | |  | | |
| **2^nd^ antibodies** | | | | **Application** | | |
| Antigen | Species | catalog No | company | dilution | comment | |
| α rb FITC | swine | #F0205 | DAKO | 1:50 | 1 hr, RT | |
| α ch AF647 | goat | #SA5-10073 | LifeTech. | 1:300 | 1 hr, RT | |
| α ms AF594 | goat | #A11032 | LifeTech. | 1:300 | 1 hr, RT | |
|  | | | |  | | |
| **directly labelled antibodies** | | | | **Application** | | |
| Antigen | Species | catalog No | company | dilution | | comment |
| S100B-FITC* | rabbit | #Z0311 | DAKO | 1:50 | | 20 min, 4°C, perm |
| CD80-PerCP-eF710 | mouse | 46-0809-42 | eBioscience | 1:25 | |  |
| CD276(B7-H3)-PE | mouse | 565829 | BD Bioscience | 1:50 | |  |
| CD40-PerCP | mouse | Ab91282 | Abcam | 1:5 | |  |
| MHCII-APC-ef780 | mouse | 47-9956-42 | eBioscience | 1:50 | |  |
| PD-L1-PE | mouse | 557924 | BD Bioscience | 1:5 | |  |
| HVEM-A647 | mouse | 564411 | BD Bioscience | 1:25 | |  |
| CD86-APC | mouse | 555660 | BD Bioscience | 1:25 | |  |
| CD273- PE | mouse | 565829 | BD Bioscience | 1:25 | |  |
| CD58-APC | mouse | 17-0578-41 | eBioscience | 1:25 | |  |

**Supplementary Table 1 │ List of antibodies.**

perm = permeabilization necessary, RT = room temperature.
