# Supplementary figures and images for: Human repair‐related Schwann cells adopt functions of antigen‐presenting cells in vitro
Source: Glia. 2022 Aug 17;70(12):2361–77. doi: 10.1002/glia.24257 (PMC9804420; doi:10.1002/glia.24257)

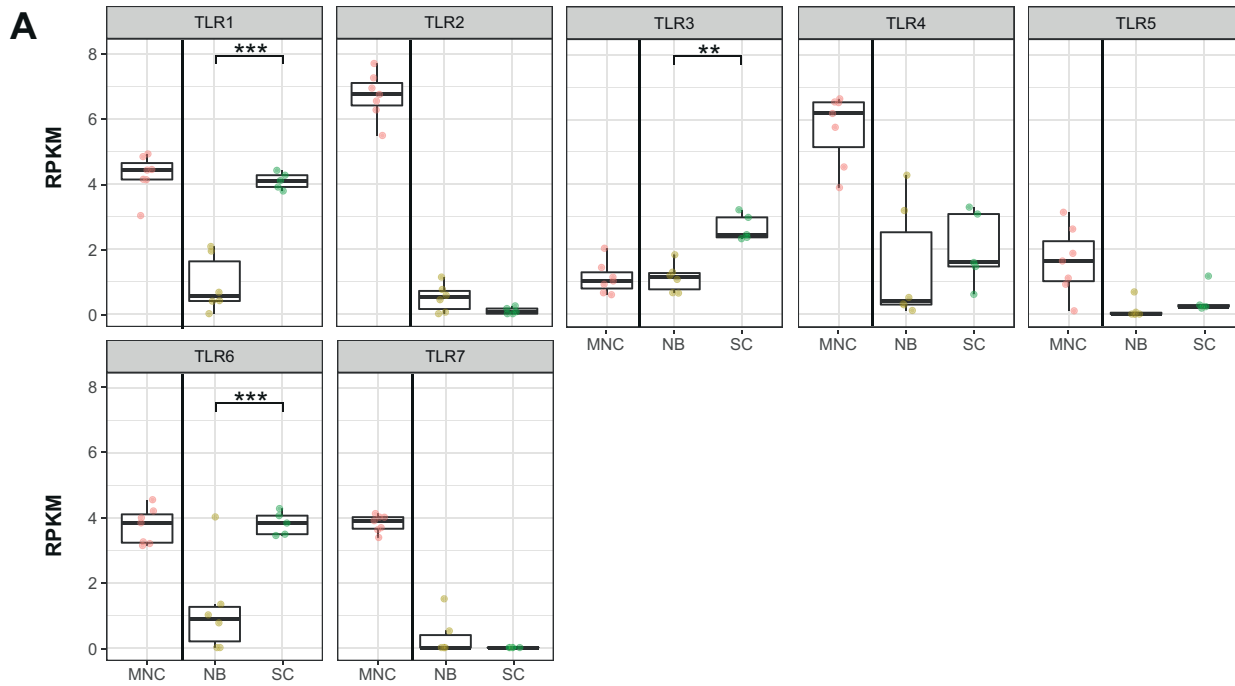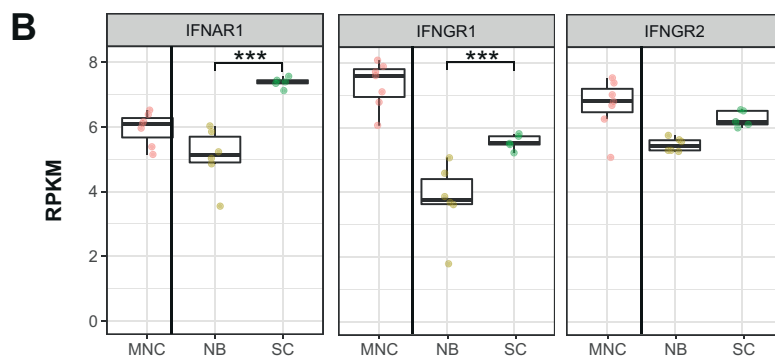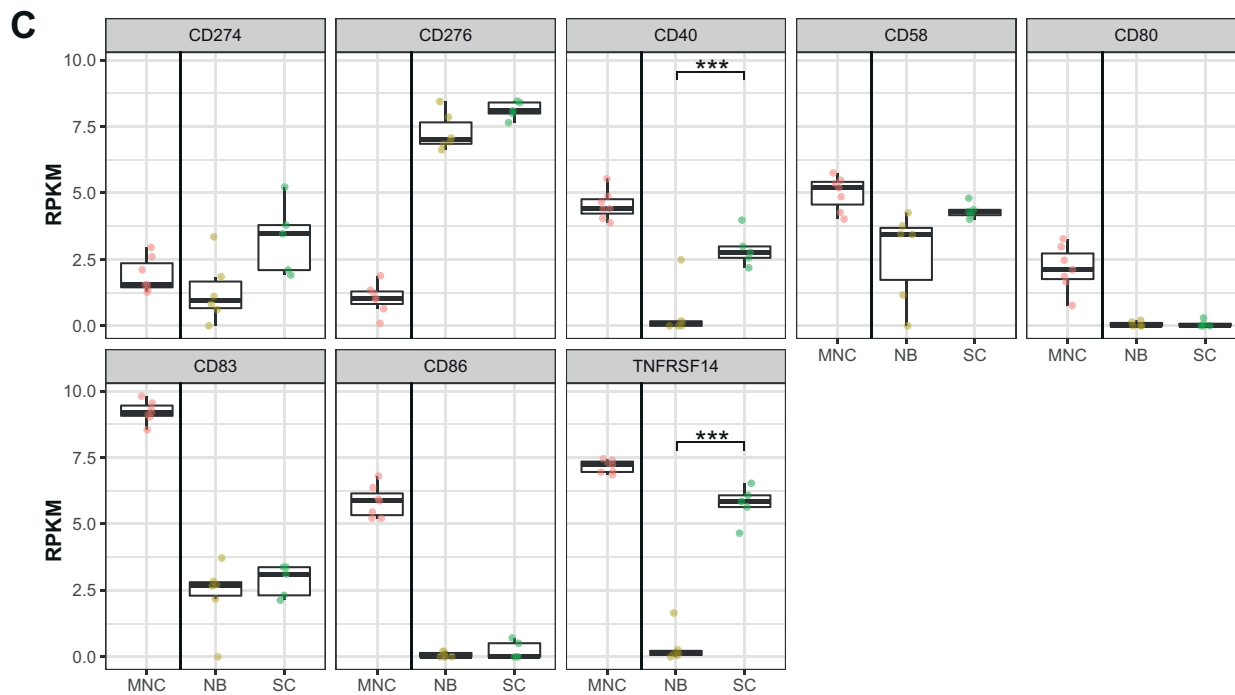

Supplement: Supplementary file 2 — Supplementary Figure 1 mRNA expression of Toll‐like receptors, interferon receptors and co‐signaling molecules. Boxplots show mRNA levels (RPKM) in hrSCs (n = 5) versus NB cells (n = 5). MNCs are shown as reference. (A) Toll‐like receptors (B) interferon receptors and (C) co‐stimulatory and inhibitory molecules. Boxes contain 50% of data and whiskers the upper and lower 25% means are displayed as black horizontal lines. [file GLIA-70-2361-s003.pdf]

**A**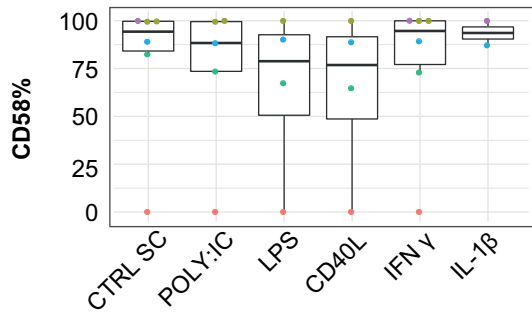**B**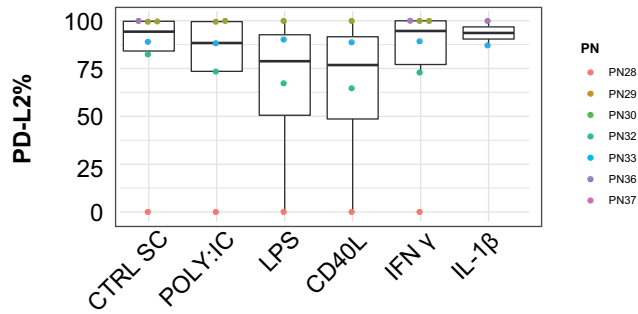**C**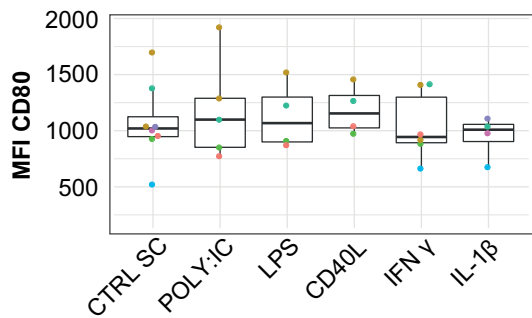**D**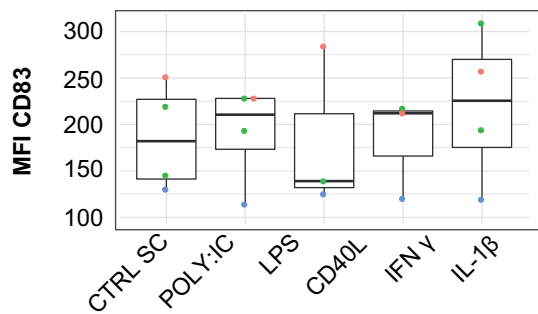**E**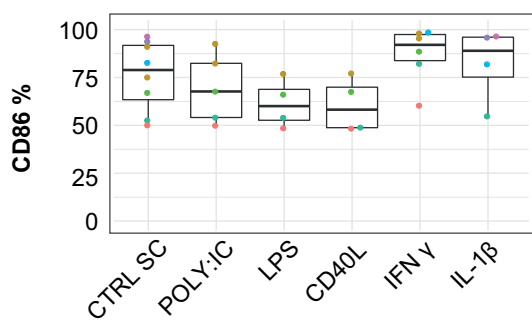**F**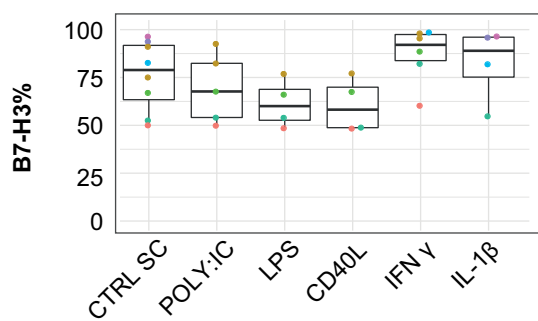

Supplement: Supplementary file 3 — Supplementary Figure 2 Flow cytometry‐based phenotyping of MHCII and co‐signaling molecules upon inflammatory stimulation. Box plots show the expression status of CD58 (A), PDL2 (B), CD80 (C), CD83 (D), CD86 (E) and B7H3 (F) of S100 positive hrSCs after stimulation with POLY:IC, LPS, CD40L, IFNγ and IL‐1β; n = 9; technical replicates (same color); biological replicates (different color). Each biological replicate is conducted with hrSCs isolated from a different donor nerve. (A, B, E, and F) Boxplots represent the percentage of positive cells based on gates set in relation to unstained controls. (C, D) Boxplots represent the mean fluorescence intensity (MFI). Boxes contain 50% of data and whiskers the upper and lower 25%; means are displayed as black horizontal lines. A two‐way ANOVA using a post‐hoc Holm p‐value correction was performed; *p ≤ 0.05; **p ≤ 0.01; and ***p ≤ 0.001. [file GLIA-70-2361-s001.pdf]

**A**

**T cell monocyte proliferation / activation day 2**

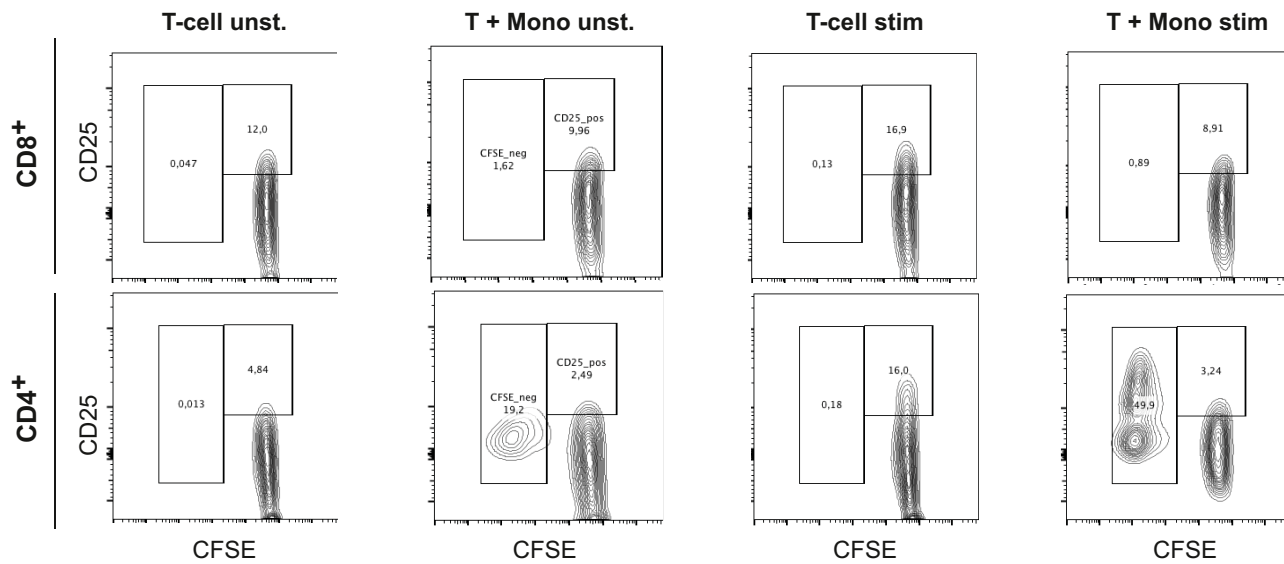

**B**

**T cell monocyte proliferation / activation**

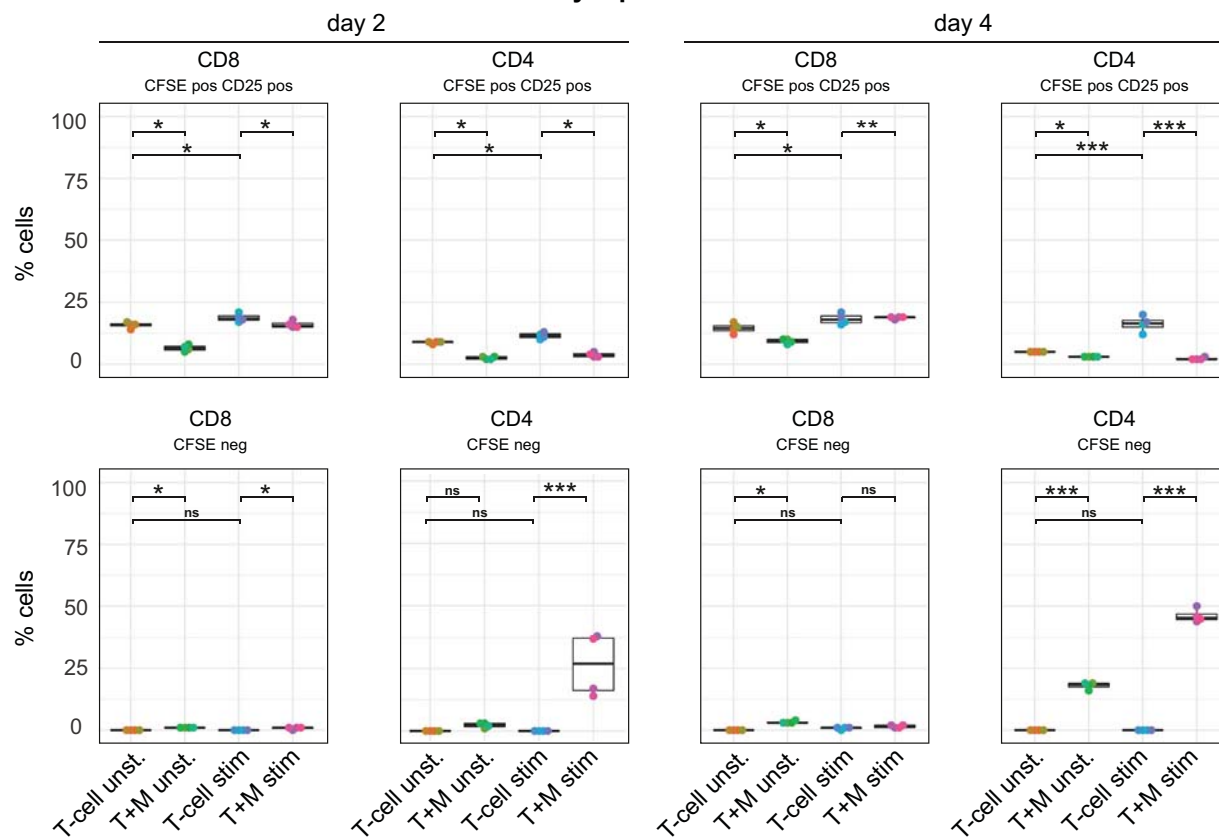

Supplement: Supplementary file 4 — Supplementary Figure 3 T‐cell activation and proliferation in co‐cultures with human peripheral blood monocytes. Co‐cultures were performed in the absence or presence of CD3/CD28 activation. (A) Representative FACS plots showing CD25 expression against CFSE of T‐cells at day 2 of co‐cultivation and in control cultures. (B) Boxplots show the CFSE+/CD25+ and CFSE− CD4+ and CD8+ T‐cells at day 2 and day 4 based on gates set as illustrated in (A). Boxplots represent the percentage of positive cells based on the parental population. Boxes contain 50% of data and whiskers the upper and lower 25%; means are displayed as black horizontal lines. A two‐way ANOVA using a post‐hoc Holm p‐value correction was performed; *p ≤ 0.05; **p ≤ 0.01; and ***p ≤ 0.001. [file GLIA-70-2361-s005.pdf]

**A****T cell activation CD8 day10**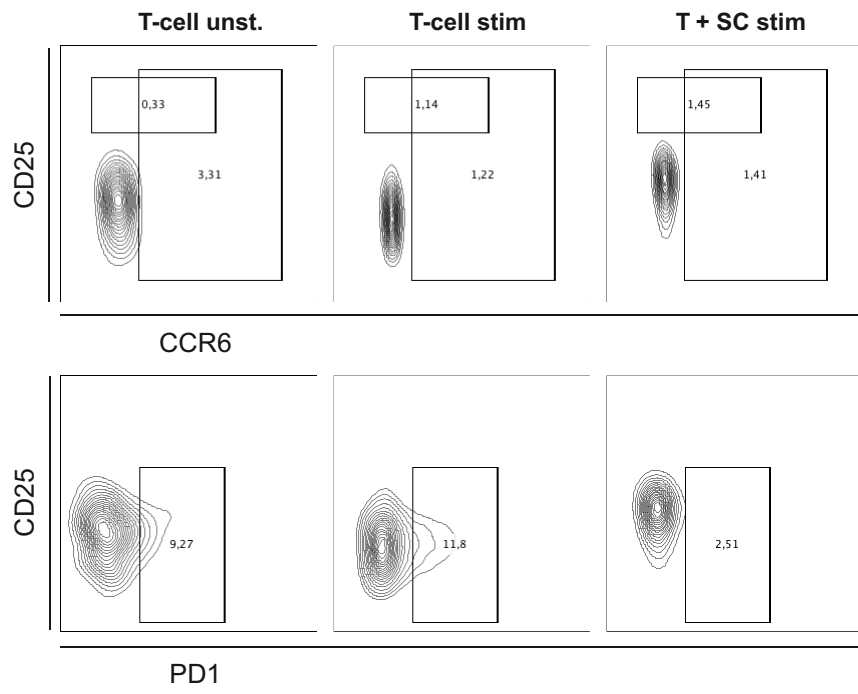**B****T cell activation CD8 day10**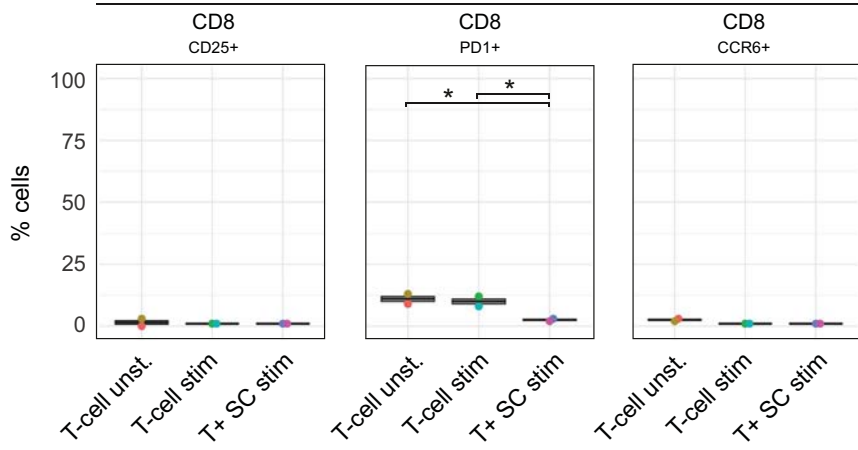

Supplement: Supplementary file 5 — Supplementary Figure 4 CD8 T‐cell subpopulations at day 10 after co‐cultivation with hrSCs and stimulation with CD3/28 beads (A) Representative FACS plots showing the gating strategy for CD25 positive, CCR6 positive and PD1 positive T‐cells after gating for CD8 positive T‐cells as illustrated in Figure 4. (B) Boxplots represent the percentage of positive cells based on the parental population. Boxes contain 50% of data and whiskers the upper and lower 25%; means are displayed as black horizontal lines. A two‐way ANOVA using a post‐hoc Holm p‐value correction was performed; *p ≤ 0.05; **p ≤ 0.01; and ***p ≤ 0.001. [file GLIA-70-2361-s002.pdf]
